# Phosphorylation of Beta-3 adrenergic receptor at serine 247 by ERK MAP kinase drives lipolysis in obese adipocytes

**DOI:** 10.1016/j.molmet.2018.03.012

**Published:** 2018-03-29

**Authors:** Shangyu Hong, Wei Song, Peter-James H. Zushin, Bingyang Liu, Mark P. Jedrychowski, Amir I. Mina, Zhaoming Deng, Dimitrije Cabarkapa, Jessica A. Hall, Colin J. Palmer, Hassan Aliakbarian, John Szpyt, Steven P. Gygi, Ali Tavakkoli, Lydia Lynch, Norbert Perrimon, Alexander S. Banks

**Affiliations:** 1 Division of Endocrinology, Diabetes and Hypertension, Brigham and Women's Hospital and Harvard Medical School, Boston, MA, 02115, USA; 2 Department of Genetics, Harvard Medical School, and Howard Hughes Medical Institute, Boston, MA, 02115, USA; 3 Department of Cell Biology, Harvard Medical School, Boston, MA, 02115, USA; 4 Department of Surgery, Brigham and Women's Hospital, and Harvard Medical School, Boston, MA, 02115, USA

**Keywords:** ERK, Beta adrenergic receptor, Lipolysis, Free fatty acid, Obesity, Insulin resistance, Adipose, Fat

## Abstract

**Objective:**

The inappropriate release of free fatty acids from obese adipose tissue stores has detrimental effects on metabolism, but key molecular mechanisms controlling FFA release from adipocytes remain undefined. Although obesity promotes systemic inflammation, we find activation of the inflammation-associated Mitogen Activated Protein kinase ERK occurs specifically in adipose tissues of obese mice, and provide evidence that adipocyte ERK activation may explain exaggerated adipose tissue lipolysis observed in obesity.

**Methods and Results:**

We provide genetic and pharmacological evidence that inhibition of the MEK/ERK pathway in human adipose tissue, mice, and flies all effectively limit adipocyte lipolysis. In complementary findings, we show that genetic and obesity-mediated activation of ERK enhances lipolysis, whereas adipose tissue specific knock-out of ERK2, the exclusive ERK1/2 protein in adipocytes, dramatically impairs lipolysis in explanted mouse adipose tissue. In addition, acute inhibition of MEK/ERK signaling also decreases lipolysis in adipose tissue and improves insulin sensitivity in obese mice. Mice with decreased rates of adipose tissue lipolysis *in vivo* caused by either MEK or ATGL pharmacological inhibition were unable to liberate sufficient White Adipose Tissue (WAT) energy stores to fuel thermogenesis from brown fat during a cold temperature challenge. To identify a molecular mechanism controlling these actions, we performed unbiased phosphoproteomic analysis of obese adipose tissue at different time points following acute pharmacological MEK/ERK inhibition. MEK/ERK inhibition decreased levels of adrenergic signaling and caused de-phosphorylation of the β3-adrenergic receptor (β3AR) on serine 247. To define the functional implications of this phosphorylation, we showed that CRISPR/Cas9 engineered cells expressing wild type β3AR exhibited β3AR phosphorylation by ERK2 and enhanced lipolysis, but this was not seen when serine 247 of β3AR was mutated to alanine.

**Conclusion:**

Taken together, these data suggest that ERK activation in adipocytes and subsequent phosphorylation of the β3AR on S247 are critical regulatory steps in the enhanced adipocyte lipolysis of obesity.

## Highlights

• Obese mice have increased ERK activity in white adipose tissues.
• Blocking ERK reduces lipolysis in flies, in mice and in human adipose tissue.
• Genetic activation of ERK promotes lipolysis.
• Identification of *in vivo* ERK substrates by proteomics in obese adipose tissue.
• ERK phosphorylates β3AR at Ser247 to enhance lipolysis in obesity.

## Graphical abstract

**Figure:**
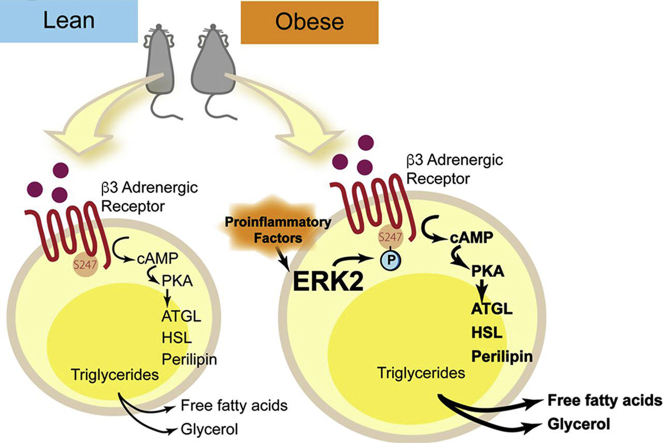

## 1 Introduction

The effects of obesity contribute to decreased life expectancy. The risks of cardio-metabolic diseases and cancer, among numerous other co-morbidities, are increased by obesity [1], [2], [3]. Mechanisms linking increased fat mass in obese individuals to the development of insulin resistance include augmented release of free fatty acids (FFA), certain hormones, and pro-inflammatory factors [4]. However, a key unanswered question remains. What drives the increased rate of basal lipolysis observed in obese adipose tissue? We observed that treating obese mice with an FDA-approved whole body MEK inhibitor dramatically improved insulin sensitivity and sought to better understand the causal mechanisms [5]. Through these studies, we investigate the relationship between obesity, increased ERK kinase activation, and stimulation of beta adrenergic-mediated lipolysis.

Circulating FFA levels are predominantly derived from stored triglycerides that are actively broken down through enzymatic lipolysis in WAT. Signals that promote lipolysis include catecholamines and natriuretic peptides. The catalytic steps responsible for catecholamine-mediated lipolysis involve the β-adrenergic activation of cAMP-dependent protein kinase (PKA), and its downstream phosphorylation targets: hormone-sensitive lipase (HSL), adipose triglyceride lipase (ATGL) and perilipin [6], [7]. Multiple lines of evidence demonstrate that obesity increases rates of basal, unstimulated lipolysis—an effect that can be reversed by weight loss surgery or thiazolidinedione treatment [8], [9], [10]. FFA released from hypertrophied obese adipose tissue have systemic effects on whole body metabolism [11]. Elevated circulating FFA promote ectopic lipid deposition in peripheral tissues, decrease glucose uptake and oxidation, increase insulin resistance, and lead to lipotoxicity induced impairment of insulin secretion from β-cells [11]. Given the strong link between obesity, lipolysis, and metabolic dysfunction, the pharmacological inhibition of lipolysis is a promising target to combat lipotoxicity [12]. Importantly, anti-lipolytic agents that lower FFA levels have potent antidiabetic effects [10], [13], [14]. Thus, identification and pharmacological inhibition of the signals driving basal lipolysis holds great therapeutic promise to counter the health burden of obesity-related disease.

The mitogen-activated protein kinase kinase/extracellular signal–regulated kinase (MEK/ERK) signaling pathway is an evolutionarily conserved signaling module that is involved in a wide variety of cellular processes including proliferation, inflammation, and metabolism [15], [16], [17]. Humans and mice express two similar ERK isoforms, ERK1 and ERK2. In mice, the *in vivo* effects of ERK1 and ERK2 are more nuanced owing to their 83% amino acid identity and biochemical redundancy [18], [19]. Although they are encoded by distinct genes [20], relative levels of ERK1 and ERK2 vary considerably from one tissue to another [21]. As a model organism with a powerful genetic toolkit and similar physiological regulation to mammals, *Drosophila* expresses a single ERK ortholog, encoded by *Rolled* (*rl*) [22]. Previous studies have indicated a conserved MEK/ERK signaling pathway and its impact on organ development and stress responses [23].

The metabolic roles of ERK in adipocyte proliferation, differentiation, and insulin action have been well studied [24]. However, the pattern of ERK dysregulation in different tissues involved in metabolic regulation and how these contribute to energy overload-associated metabolic dysfunction remains controversial. Here we show that obesity increases ERK kinase activity levels in adipose tissue and fat body from mice and Drosophila, respectively. We find that mouse primary adipocytes selectively express ERK2 but have undetectable levels of ERK1. Adipocyte-specific ERK2 knock-out mice (ERK2AKO) and fat body-specific ERK knockdown flies exhibit decreased rates of lipolysis. We find that *in vivo* inhibition of the MEK/ERK pathway alters lipolysis in adipose tissue, by decreasing β3AR phosphorylation at serine 247 and subsequent downstream phosphorylation events that control release of FFA. Mice with ERK inactivation or inhibition also fail to appropriately activate thermogenesis and defend body temperature upon cold challenge due to lack of substrate availability. We conclude that ERK plays a critical role in regulating lipolysis from obese adipose tissue through its direct phosphorylation of β3AR, and these are likely contributing mechanisms to insulin resistance and type 2 diabetes.

## 2 Materials and methods

### 2.1 Chemicals and antibodies

CL-316,243 (149910) was purchased from Thermo Fisher Scientific. GSK1120212/Trametinib (S2673) and PD0325901 (S1036) were purchased from SelleckChem. Atglistatin (SML 1075) and 8-Br-cAMP (B5386) were purchased from Sigma–Aldrich. NEFA detection kit (999–34691, 995–34791, 991–34891, 993–35191, 276–76491) and Triglyceride detection kit (461–08992, 461–09092) were purchased from Wako Diagnostics. Rabbit anti-pERK (9101 for mouse and 4370 for *Drosophila*), rabbit anti-ERK (4695), rabbit anti-pAKT (13038), rabbit anti-AKT (9272), rabbit anti-pHSL (4139P), rabbit anti-HSL (4107P), rabbit anti-MapK/CDK Substrate (2325), rabbit anti-β-Tubulin (2146), and HRP-conjugated secondary antibodies against rabbit (7074S) and mouse (7076S) were obtained from Cell Signaling Technologies. Rabbit anti-β3AR (sc-515763) and rabbit anti-HA tag (sc-805) antibodies were purchased from Santa Cruz Biotechnology. Mouse anti-actin (ab3280) antibody was purchased from Abcam. Mouse anti-FLAG tag (F1804) antibody was purchased from Sigma–Aldrich. HA antibody-conjugated agarose (PI26181) was purchased from Thermo Fisher Scientific.

### 2.2 Plasmids

HA-tagged mouse β3AR coding sequence was amplified by PCR from pGL2-HA-mβ3AR construct, a kind gift from Dr. Sheila Collins [25] and cloned into pcDNA3.1 vector. Ser247-mutated β3AR construct was generated by QuikChange site-directed mutagenesis kit from Agilent Technologies (#200519) based on pcDNA3.1-HA-mβ3AR. CRISPR/Cas9 vector (PX459) was ordered from Addgene (Plasmid #48139). β3AR-targeted Crispr sgRNAs (sense: 5′-CACCGGGCAGAGTCCACCGCTCAAC-3’; antisense: 5′-AAACGTTGAGCGGTGGACTCTGCCC-3′) were synthesized by Integrated DNA Technologies and cloned into PX459 following the protocol provided by Dr. Feng Zhang [26]. Constitutively active ERK2 (ERKca) expression construct was previously described [27].

### 2.3 Drosophila

Flies were maintained at 25 °C on a 12 h light, 12 h dark light cycle. Flies were raised on a standard cornmeal/agar diet (10 g/L agar, 230 g/L soy flour, 30 g/L yeast, 100 g/L cornmeal, 74 g/L molasses, 4.5 g/L propionate, and 6 g/L Nipogen). 25% extra sucrose was added into the standard diet to generate the high-sugar diet. For high-sugar feeding, the parental females were allowed to lay eggs on high-sugar food media, such that larvae were cultured on high-sugar food throughout development. All experiments were performed using 3rd instar stage larvae (12 h before wandering). *Cg-GAL4* was used to target the larval fat body. *UAS-rl*^*Sem*^ (BLM 59006) and *UAS-rl-RNAi* (DRSC HMS00173) were crossed to Cg-GAL4 to generate overexpression of a constitutively active ERK and knockdown of ERK in larval fat body, respectively. Circulating carbohydrate and glycerol measurements were previously described [28], [29]. Briefly, 2 μL hemolymph from 8 3rd instar larvae was diluted in 38 μL sugar buffer (5 mM Tris pH 6.6, 137 mM NaCl, 2.7 mM KCl) and heated at 70° to inactivate endogenous enzymes. After centrifugation at 14,000 g for 10 min, 10 μL supernatant was treated with 0.2 μL water or trehalase (Megazyme, E-TREH) at 37 °C for 20 min to digest trehalose into glucose, and glucose was measured by incubation with 150 μL D-Glucose Assay reagent (Megazyme, K-GLUC) at 37 °C for 5 min. The absorbance at 510 nm was measured on a SpectraMax Paradigm plate reader. The circulating trehalose levels were determined by subtracting the circulating free glucose levels from the total glucose levels after enzyme digestion. Circulating glycerol was measured by incubating 10 μL supernatant with 100 μL Glycerol Reagent (Sigma, F6428). TAG measurement was performed as previously described [29], [30]. Briefly, 5 larval fat bodies were homogenized in 500 μL 0.1% Triton-X 100/PBS, heated at 70 °C for 5 min, and centrifuged at 14,000 g for 10 min. 10 μL supernatant was used to measure TG using Serum TG determination kits (Sigma, TR0100). Protein amounts were measured using Bradford Reagent (Sigma, B6916). TAG storage was normalized to protein amount. Neutral lipids in larval fat body were visualized after dissection, fixation for 15 min in 4% formaldehyde/PBS. After fixation, the samples were washed with 0.2% Triton-X 100/PBS and incubated with Bodipy 493/503 (Thermo Fisher Scientific, D3922. 1 mg/mL) and DAPI (Sigma, 1:1000) were used for neutral lipid and nucleus staining, respectively, for 30 min at room temperature.

### 2.4 Mice

ERK2^flox/flox^ (also known as Mapk1^flox/flox^) mice were obtained from Jackson Labs (019112). ERK2^flox/flox^ mice were bred with adipocyte-specific Adipoq-Cre1Evdr/J (Jackson Labs, 028020) to generate mice with adipocyte-specific deletion of ERK2. Routine genotyping for Adiponectin Cre, wild type, and floxed ERK2 was performed as described on the Jackson Labs website. In this study, wild type control mice (WT) are ERK2^flox/flox^ genotype, while ERK2AKO mice are Adipoq-Cre^+^::ERK2^flox/flox^. For chow vs HFD studies in wild type mice, C57BL/6J mice were purchased from Jackson Laboratories (Jackson Labs, 000664). The standard chow diet was purchased from PicoLab (PicoLab Rodent Diet 20, #5053). HFD with 60% of calories from fat was obtained from Research Diets (Research Diets, D12492, irradiated). Mice were fasted for 4 h prior to sacrifice. All animal studies were approved by the Brigham and Women's Hospital IACUC.

### 2.5 Human adipose tissue

Institutional Review Board approval and informed patient consent were obtained prior to collecting subcutaneous adipose tissue from the abdominal wall area during laparoscopic Roux-En-Y gastric bypass surgery.

### 2.6 Mice treated with MEKi and ATGLi

C57BL/6J mice were purchased from Jackson Laboratories (Jackson Labs, 000664) and fed with HFD for 8 weeks. Chronic administration: Trametinib (MEKi, 3 mg/kg) or PD0325901 (PD, 10 mg/kg) was administered by daily oral gavage for 5 days. Compounds were dissolved in DMSO and diluted into an aqueous 250 μL dose containing 0.5% hypromellose and 2% Tween-80. High-fat diet mice were switched to and maintained on a standard chow diet 48 h before the first dose of MEK inhibitors. The mice were then fasted overnight. Sera from the tail vein were collected and assayed for fatty acid levels. For acute administration and cold exposure experiments, mice were implanted with intraperitoneal telemetry temperature probes one week before the experiments. On the day of the experiment, mice were given MEKi (3 mg/kg) or ATGLi (200 μmol/kg) by i.p. injection at 9 am. The temperature transition from 30 °C to 4 °C was performed at 12 pm and over a period of 3 h. The body temperature of each mouse was reported at 5pm, and serum was collected from the tail between 5 pm and 5:30 pm. For olive oil rescue experiments, mice were given MEKi (3 mg/kg) by i.p. injection at 9 am in the morning and then gavaged with 5 mL/kg of PBS or olive oil at 11:30 am. The gradual temperature transition from 30 °C to 4 °C was performed at 12 pm, and the body temperature of each mouse were reported at 5pm. Then, serum was collected at between 5 pm and 5:30 pm and analyzed for fatty acid levels.

### 2.7 Indirect calorimetry

Chow diet fed and HFD fed control and ERK2AKO male mice were implanted with intraperitoneal telemetry temperature probes one week before the beginning of indirect calorimetry experiments. For indirect calorimetry, following a 24-hour acclimation period, mice were housed at thermoneutrality (30 °C) prior to cold exposure. The temperature transition from 30 °C to 4 °C was performed over a period of 3 h. Oxygen consumption, carbon dioxide production, food consumption, movement, and energy expenditure were measured using CLAMS apparatus (Columbus Instruments) available to the Brigham and Women's Hospital Metabolic Phenotyping Core. Statistical analysis and plotting were performed in the R programming language [31] with *CalR*, a freely available web resource with a graphical user interface for analysis of indirect calorimetry using analysis of covariance [32].

### 2.8 Generation of β3AR null 3T3-L1 cell lines by CRISPR/Cas9

CRISPR/Cas9 constructs with sgRNA targeted to the β3AR gene were transfected into 3T3-L1 with lipofectamine 2000 (Invitrogen, #11668-019). Transfected cells were then selected with puromycin for 5 days. Surviving cells were re-plated into 96-well plates at density of 0.5 cell per well for clonal limiting dilution. Colonies grown from single cells were initially screened by high throughput High Resolution Melt-Curve Analysis. Positive colonies were confirmed for gene editing by Sanger sequencing and expanded for further experiments. More than 200 clones were screened for knock-in of Adrb3 S247A by homology directed repair in 3T3-L1 cells, without success.

### 2.9 Statistical analysis

Comparisons for statistical significance performed by one-way or two-way ANOVA as indicated, followed by Holm-Sidak post hoc testing unless otherwise specified.

Additional methods can be found in the supplementary online materials.

## 3 Results

### 3.1 Obesity increases ERK phosphorylation specifically in white adipose tissue

**Figure 1:**
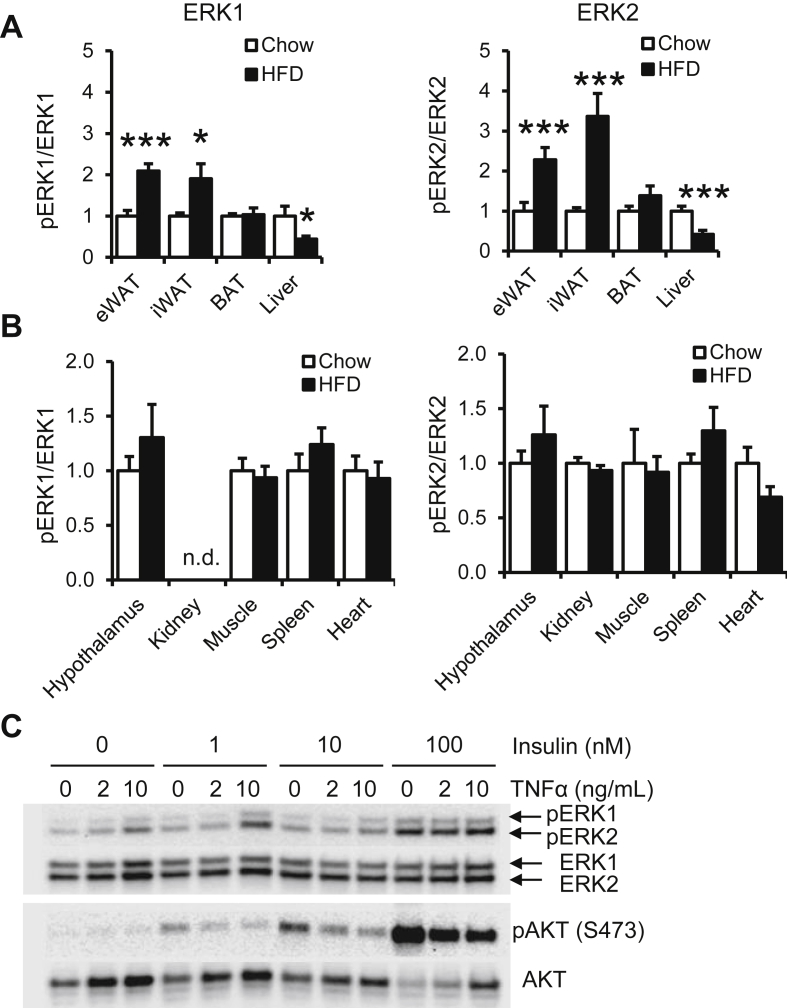
**Effect of obesity and insulin resistance on ERK phosphorylation.** Quantitation of phosphorylated ERK1 and ERK2 relative to total ERK1 and ERK2 in (A) epididymal white adipose tissue (eWAT), inguinal white adipose tissue (iWAT), brown adipose tissues (BAT), and liver. (B) pERK/ERK levels in hypothalamus, kidney, muscle (quadriceps), spleen, and heart from age-matched 16 week old chow fed and HFD fed (8 weeks on HFD) mice. Mice were fasted for 4 h prior to sacrifice. (C) 3T3-L1 adipocytes were treated with 0 ng/mL, 2 ng/mL, or 10 ng/mL TNFα for 4 days, followed by treatment of 0 nM, 1 nM, 10 nM or 100 nM insulin. The protein lysates were blotted for phosphorylated ERK (pERK1/2), total ERK (ERK1/2), phosphorylated AKT at S473 (pAKT(S473)) and total AKT (AKT). Error bars represent SEM. Statistical significance was analyzed by Student's t-test. *, p < 0.05; ***, p < 0.005, n.d., not detected.

During obesity, adipose tissue expansion leads to inflammatory cell recruitment, tissue inflammation, and secretion of inflammatory cytokines. ERK phosphorylation increases in the adipose tissue of obese leptin-deficient *ob/ob* mice [5]. However, it is unknown whether this phenomenon is specific to adipose tissue or occurs more globally. To investigate this question, we examined ERK1 and ERK2 phosphorylation in tissues from age-matched mice fed a standard low-fat chow or an obesogenic 60% high fat diet. We observed increased ERK phosphorylation in epididymal and inguinal white adipose tissues but not in brown adipose tissues, and decreased ERK phosphorylation in livers from Diet-induced obesity (DIO) mice (Figure 1A and Supplementary 1A). We also observed no change in hypothalamus, kidney, muscle, spleen, or heart (Figure 1B and Supplementary 1B). These results suggest that elevated ERK activity is not a global phenomenon in obesity but occurs in a tissue-specific manner.

### 3.2 Proinflammatory cytokine-mediated signaling increases ERK activation and decreases AKT signaling in adipocytes

To understand the effect of elevated adipose-specific ERK phosphorylation levels, we examined the response of adipocytes to proinflammatory cytokines and insulin stimulation, both of which induce ERK activation [33], [34]. We chose the pro-inflammatory cytokine TNFα, a well-characterized inducer of cellular insulin resistance [35], [36]. We investigated whether ERK activation could impact canonical insulin-stimulated activation of IRS/PI3K/AKT phosphorylation. In differentiated 3T3-L1 adipocytes, we confirm that in isolation, either insulin or TNFα produce dose-dependent ERK activation. Pre-treatment with TNFα induced insulin resistance, indicated by reduced AKT S473 phosphorylation in response to insulin. However, TNFα treatment does not reduce levels of ERK phosphorylation in response to insulin. These findings suggest a model of selective insulin signaling. Pro-inflammatory cytokines decrease activation of the IRS/PI3K/AKT pathway but not the Ras/Raf/MEK/ERK pathway. These findings support a model in which the inflammation and hyperinsulinemia of obesity may both contribute to elevated ERK activation in adipose tissue (Figure 1C and Supplementary Fig. 1C).

### 3.3 Systems proteomics identifies changes in phosphorylation on key components in the β3AR signaling pathway upon MEK/ERK inhibition

**Figure 2:**
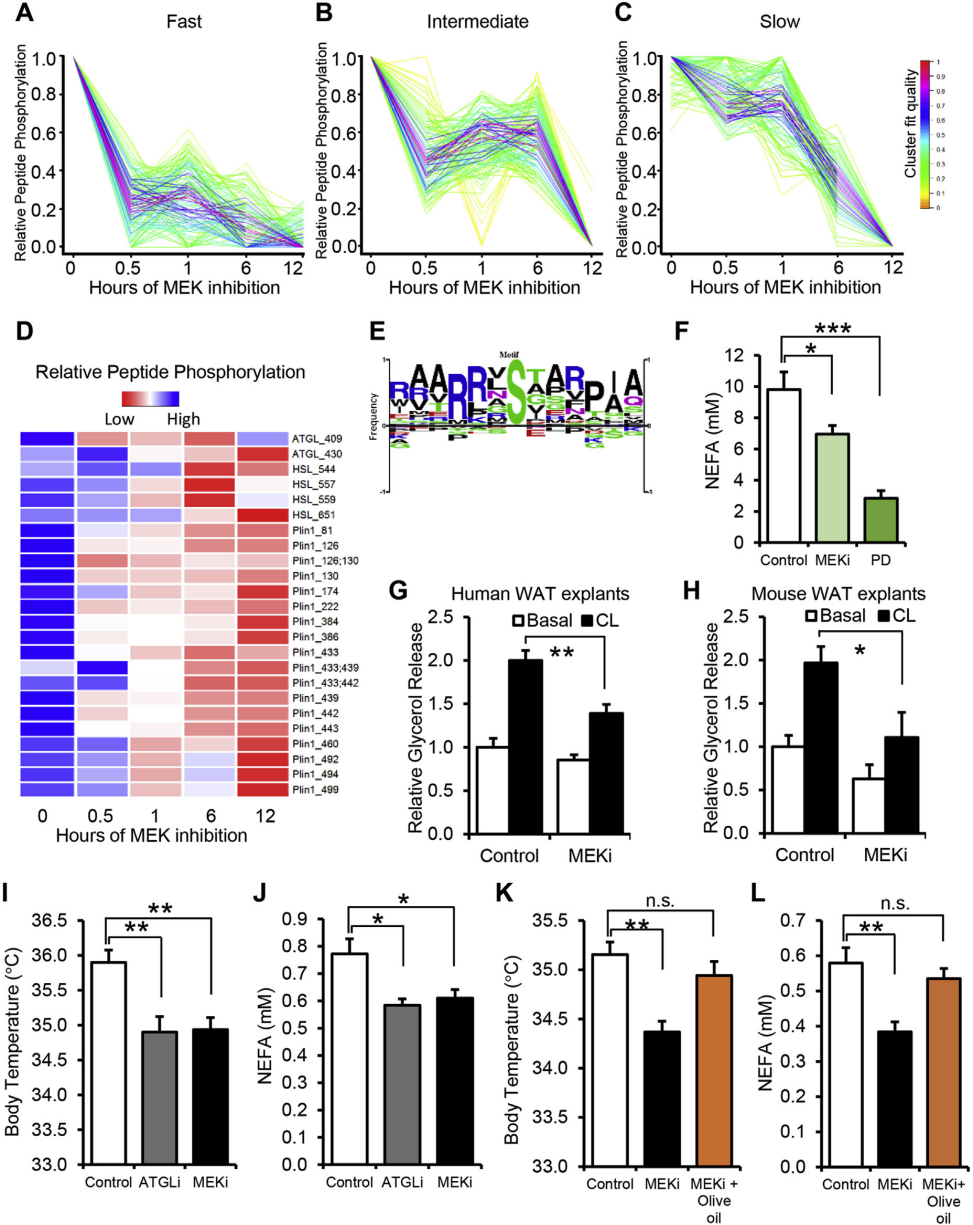
**MEK inhibition impairs β3AR signaling, lipolysis and thermogenesis**. (A–C) Temporal clustering of quantitative mass spectrometry on WAT phosphopeptides from diet induced obese mice following MEKi treatment for 0, 0.5, 1, 6 or 12 h (mean of n = 2 per group). Each line represents a unique peptide; line color represents how well a peptide fits within the cluster. (D) Heat map of peptides' relative phosphorylation from proteins controlling adipocyte lipolysis. (E) Sequence logo from peptides in 2D showing a PKA consensus phosphorylation motif (RRxS). (F) Non-esterified fatty acid (NEFA) levels in plasma fom obese mice treated with MEK inhibitors Trametinib (MEKi) or PD0325901 (PD) (n = 7–9 per group) (G) Effect of MEKi on adipose tisse explants from human subcutaneous adipose tissue in a glycerol release assay. (H) Effect of MEKi on adipose tissue explants from mouse inguinal WAT in a glycerol release assay (I) Core body temperature measured by subcutaneous telemetry probes and (J) NEFA in mice treated with saline (Control), ATGL inhibitor (ATGLi) or MEKi during a 4 °C cold temperature challenge. (K) Core body temperature and (L) NEFA in mice treated with vehicle (Control), MEKi, or MEKi and exogeneous fatty acids (Olive oil) during a 4 °C cold temperature challenge (n = 8 per group). Error bars represent SEM. Statistical significance was analyzed by one-way ANOVA n.s., р > 0.05; *, р < 0.05; **, p < 0.001; ***, p < 0.005.

To identify the proteins affected by the increased ERK phosphorylation observed in obese adipose tissue, we performed phosphoproteomic analysis of obese mouse epididymal WAT following *in vivo* administration of MEK inhibitor GSK1120212/Trametinib/Mekinist (MEKi) for 0, 0.5, 1, 6, and 12 h prior to tissue harvest. While more than 9,000 phosphorylated residues from WAT were quantified by multiplexed-based phosphoproteomics, the phosphorylation of most proteins was unchanged by MEK inhibition. We performed temporal clustering of peptides similarly affected by MEK inhibition. We focused our attention on the clusters containing proteins with decreased phosphorylation with fast, intermediate, or slow kinetics (Figure 2A–C). Lipolysis-related phosphopeptides were over-represented in the fast cluster. These included perilipin, hormone sensitive lipase (HSL/*Lipe*), and adipose triglyceride lipase (ATGL/*Pnpla2*) (Figure 2D). The phosphorylation motifs from these peptides did not contain the consensus ERK (Px_0-2_[S/T]P) kinase phosphorylation motif. Instead, they strongly represent known substrates of cAMP-dependent Protein Kinase/Protein Kinase A (PKA) with the consensus motif ([R/K][R/K]x[S/T]) (Figure 2E). The specificity of Trametinib has been tested against a panel of over 180 kinases and found to have negligible inhibitory activity on PKA [37]. Indeed, most phosphopeptides in these clusters contain ERK and not PKA phosphorylation motifs (Supplementary Fig. 2). These findings suggest that ERK indirectly regulates cAMP-PKA activation, rather than directly phosphorylating lipolytic enzymes.

### 3.4 Pharmacological inhibition of ERK phosphorylation suppresses lipolysis and thermogenesis

We next sought to determine whether the phosphoproteomic findings of decreased pro-lipolytic signaling have physiological significance. We investigated whether pharmacological inhibition of MEK/ERK signaling suppresses lipolysis. DIO wild type C57BL/6J mice were treated with two different inhibitors of MEK, PD0325901 (PD) and Trametinib (MEKi), both of which suppress the phosphorylation and activation of ERK. Our data show that MEK inhibitors significantly decreased FFA in plasma from overnight fasted animals, suggesting reduced *in vivo* lipolysis (Figure 2F). We also tested the effects of MEK inhibitors on lipolysis in an *ex vivo* model by pretreating adipose explants and examining glycerol release. We found that the pretreatment of human subcutaneous or mouse inguinal adipose explants with MEKi blocked CL induced lipolysis (Figure 2G, H). In response to cold ambient temperatures, mammalian lipolysis is required to fuel a thermogenic response to defend core body temperature [38]. To test if ERK-mediated lipolysis is necessary for the thermogenic response, we monitored body temperature of mice receiving oral vehicle control, MEK inhibitor, or a known inhibitor of lipolysis, Atglistatin (ATGLi). Both MEK inhibitor and Atglistatin-treated mice had lower body temperature and serum FFA at 4 °C (Figure 2I,J) compared to control mice. Giving exogenous FFA (olive oil) before the cold challenge prevented the decrease in body temperature and restored serum FFA levels observed with MEK inhibitor treatment (Figure 2K,L). These data suggest that pharmacological MEK inhibition reduces lipolysis *in vivo*.

### 3.5 Genetic control of ERK regulates lipid metabolism in Drosophila

**Figure 3:**
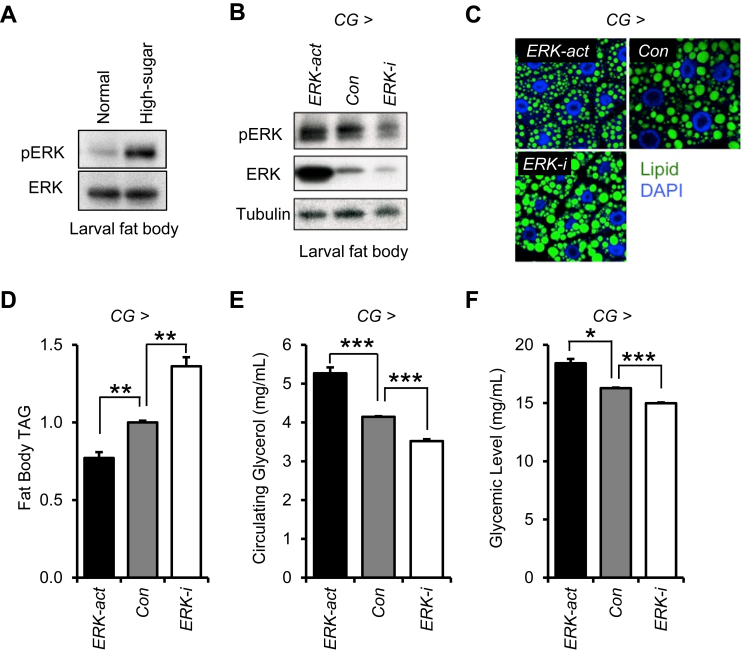
**Regulation of lipolysis in Drosophila by ERK.** (A) Phosphorylated ERK (pERK) and total ERK (ERK) in larval fat body from standard diet fed Drosophila (Normal) and high-sugar diet-induced obese Drosophila (High-sugar). (B) Phosphorylated ERK (pERK), total ERK (ERK), and Tubulin levels in larval fat bodies from indicated genotypes (*Con: Cg-GAL4/*+*. ERK-act: Cg-GAL4/UAS-r^lSem^. ERK-i: Cg- GAL4/*+*; UAS-rl-RNAi/*+). (C) Staining of lipid droplets in the larval fat body from indicated genotypes. (D) The level of triacylglyceride (TAG) stored in the fat body from indicated genotypes as described above. (E, F) The circulating glycerol level (E) and glycemic level from indicated genotypes. Error bars represent SEM. Statistical significance was analyzed by one-way ANOVA. *, p < 0.05; **, p < 0.01; ***, p < 0.005.

Similar to high-fat diet (HFD) feeding in mammals, a chronic high-sugar diet causes an obesity-like phenotype of increased fat accumulation, impaired fat body insulin signaling, and hyperglycemia in Drosophila larvae [39]. Due to the ease of genetic manipulation in Drosophila and their propensity to diet-induced obesity, we used this model for initial tests of ERK's effects on lipolysis *in vivo*. Importantly, and consistent with mouse ERK activation with HFD-induced obesity, we also observed elevated ERK phosphorylation in the fat body following a high-sugar diet that causes obesity and hyperglycemia (Figure 3A). To analyze the role of ERK signaling in Drosophila fat body, we genetically manipulated ERK expression using *Cg-GAL4*, a specific driver targeting the fat body [40]. Interestingly, fat body overexpression of a constitutively-active ERK (*Cg* > *ERK-act*. Genotype: *Cg-GAL4/UAS-rl*^*Sem*^) significantly decreased lipid droplet size and triglyceride storage in the larval fat body as compared to controls (*Con.* Genotype: *Cg-GAL4/*+). Conversely, fat body knockdown of endogenous ERK (*Cg* > *ERK-i*. Genotype: *Cg-GAL4/*+; *UAS-rl-RNAi/*+) had the opposite effect, increasing lipid droplet size and triglyceride storage (Figure 3C,D). Constitutive ERK activation in the fat body increases circulating glycerol levels in hemolymph, the Drosophila blood equivalent, suggesting enhanced lipolysis (Figure 3E). Interestingly, circulating trehalose, a disaccharide form of glucose that is the major carbohydrate in fly hemolymph, is also significantly increased by fat body ERK activation (Figure 3F). Consistent with these findings, genetic deficiency of ERK in larval fat body has reciprocal lowering effects on circulating glycerol and glucose levels (Figure 3E, F). These results support a specific role for ERK in regulating lipolysis *in vivo*.

### 3.6 ERK2 but not ERK1 is expressed in mouse adipocytes

**Figure 4:**
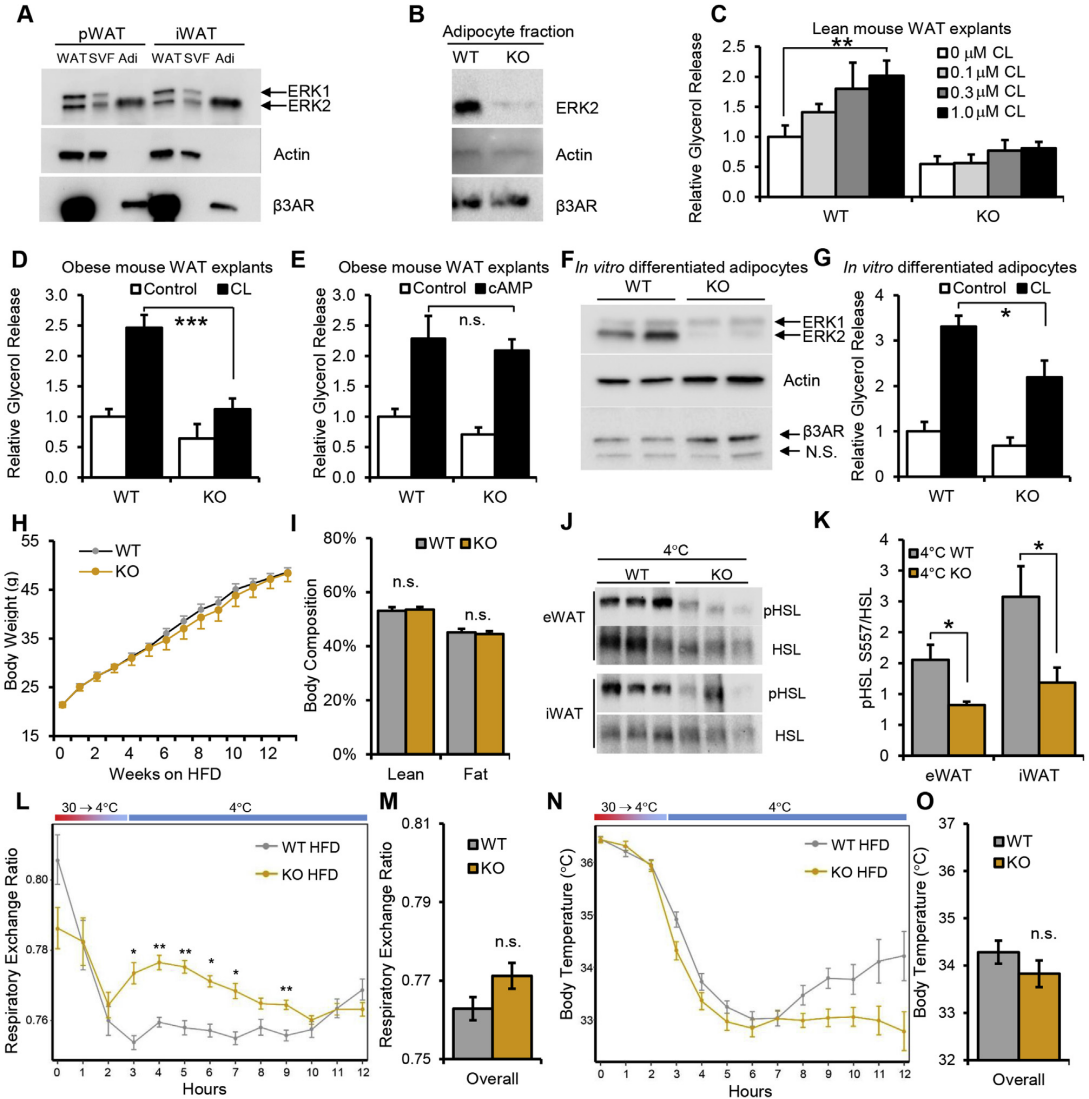
**Mouse primary adipocytes exclusively express ERK2 and genetic adipocyte ERK2 deficiency impairs lipolysis.** (A) ERK1 and ERK2 protein levels in mouse perigonadal WAT (pWAT) and inguinal WAT (iWAT). Comparison of whole tissue (WAT) and adipose tissue fractionated into SVF and floated primary adipocytes (Adi). Actin and β3AR proteins are preferentially expressed in SVF and adipocytes respectively. (B) ERK2 protein expressed in floated primary adipocytes isolated from wild type (WT) and ERK2AKO (KO) mice. (C) iWAT explants from lean WT and ERK2AKO (KO) mice were treated with 0 μM, 0.1 μM, 0.3 μM and 1 μM of selective β3AR agonist CL-316,243 (CL). (D,E) Glycerol release in iWAT explants from obese wild type (WT) and ERK2AKO (KO) mice treated with (D) 1 μM CL or (E) 0.5 mM 8-Br-cAMP (cAMP). ERK1 and ERK2 protein expression (F) and CL-induced glycerol release (G) from *in vitro* differentiated adipocytes derived from WT and ERK2AKO (KO) mice. (H) Average body weight of WT and ERK2AKO (KO) mice (n = 9 per genotype) on HFD. (I) Body composition represented as percent body fat mass and percent lean mass in WT and ERK2AKO (KO) mice on HFD (n = 9 per genotype). (J and K) Phosphorylation of HSL at Ser557 following a 3-hr cold exposure in eWAT and iWAT (J) Western blotting and (K) band quantitation analysis by one-tailed Student's t-test. (L–O) Indirect calorimetry measurements of WT and ERK2AKO mice (KO) (n = 9 per genotype) when challenged with cold exposure. The average Respiratory Exchange Ratio (L, M) and body temperatures were plotted (N, O). Error bars represent SEM. Statistical significance was analyzed by one-way ANOVA. n.s., p > 0.005; *, p < 0.005; **, p < 0.01.

To test the function of ERK in mouse adipose tissue, we first examined ERK expression in whole adipose tissue and fractionated adipose tissue—including both stromal vascular fraction (SVF) and floating adipocytes. In contrast to Drosophila, mice express two ERK isoforms, ERK1 and ERK2 (Figure 4A). Although both ERK1 and ERK2 protein appears at nearly equal levels in whole adipose tissue and SVF, ERK2 protein is the dominantly expressed isoform in fractionated adipocytes (Figure 4A). Prior studies in primary rat adipocytes have also demonstrated greater levels of kinase activity for ERK2 over ERK1 [41]. As a control for successful separation of mouse SVF and primary adipocytes, expression of β-actin and β3AR were measured (Figure 4A). Μature adipocytes have low levels of β-actin but high levels of β3AR, while the opposite pattern is observed in SVF [42], [43], [44]. To investigate the role of ERK2 in adipocytes, we generated mice with adipocyte-specific deletion of ERK2 (ERK2AKO) by crossing ERK2-floxed mice [45] with Adiponectin-Cre transgenic mice [46]. The deletion of ERK2 and absence of ERK1 and ERK2 protein in fractionated primary adipocytes but not in other tissues from ERK2AKO mice was confirmed by western blotting (Figure 4B and Supplementary Fig. 3A). Despite lacking ERK2, primary adipocytes from ERK2AKO had normal expression of β3AR, the most highly expressed adrenergic receptor in murine adipocytes (Figure 4B) and demonstrated normal levels of differentiation markers (Supplemental Figs. 3B and 3C).

### 3.7 Adipocyte ERK2 regulates lipolysis

We next examined lipolysis in this model of adipocyte-specific ERK2 deletion. Lipolysis induced by the β3AR-specific agonist CL 216,343 (CL) was blocked in adipose explants from lean ERK2AKO mice (Figure 4C). We observed similar blockade of lipolysis upon CL treatment of adipose explants from obese ERK2AKO mice (Figure 4D). Critically, we also tested whether ERK2 deletion would affect lipolysis following cAMP stimulation, which bypasses upstream β3AR signaling. Our data showed that there is no significant difference in the level of glycerol released by cAMP stimulated adipose explants from ERK2AKO mice compared with the control littermates (Figure 4E). We next examined whether the effect of ERK2 deletion on lipolysis is cell autonomous, by differentiating WT or ERK2 AKO SVF into adipocytes *in vitro* (Figure 4F and Supplementary Fig. 3C). Consistent with the data from adipose explants, we find CL-induced lipolysis was significantly impaired in ERK2-deficient adipocytes differentiated *in vitro* (Figure 4G).

### 3.8 Adipocyte ERK2 deficiency limits thermogenesis during cold challenge

Despite the deficiency of ERK2 and impaired activation of lipolysis in adipocytes, ERK2AKO mice are born at expected Mendelian ratios, are viable, fertile, and gain weight normally when fed with high fat diet (Figure 4H). There is no difference in body composition when comparing HFD ERK2AKO mice with control mice (Figure 4I). Correspondingly, we observed similar food intake and energy expenditure between WT and ERK2AKO mice on either standard chow or HFD (Supplemental Fig. 4). To examine whether cold-induced lipolysis was altered in ERK2AKO mice, we measured PKA-mediated phosphorylation of HSL, a molecular marker of active lipolysis. We observe a significant decrease in the ratio of pHSL to total HSL in WAT from ERK2AKO mice during a cold challenge (Figure 4J,K). When challenged with cold and monitored by indirect calorimetry, ERK2AKO mice had higher Respiratory Exchange Ratio (RER) compared to control littermates, suggesting decreased fatty acid oxidation as an energy source in ERK2-deficient mice (Figure 4L,M). In addition, there is a trend towards lower body temperature in cold-challenged ERK2AKO mice. These results failed to reach statistical significance as the experiment was terminated prematurely due to the rescue of two ERK2AKO mice from rapidly falling body temperature (Figure 4N,O). These observations are analogous to the results from the cold challenge experiments in the MEK inhibitor-treated mice. Lack of ERK2 in adipocytes did not affect whole-body glucose homeostasis or insulin sensitivity as measured by glucose tolerance tests and insulin tolerance tests (Supplementary Figs. 3D and 3E). However, ERK2AKO mice had increased insulin signaling in white adipose tissue but not liver (Supplemental Figs 3F and 3G) These data suggest that modest improvement in adipose tissue insulin sensitivity was not sufficient to perturb whole body glucose homeostasis. These data also suggest that genetic deletion of ERK2 in mouse adipocytes has similar consequences on lipolysis to the *in vivo* pharmacological inhibition of MEK/ERK signaling.

### 3.9 Inhibition of MEK/ERK signaling in adipocytes blocks β3AR agonist-induced lipolysis

**Figure 5:**
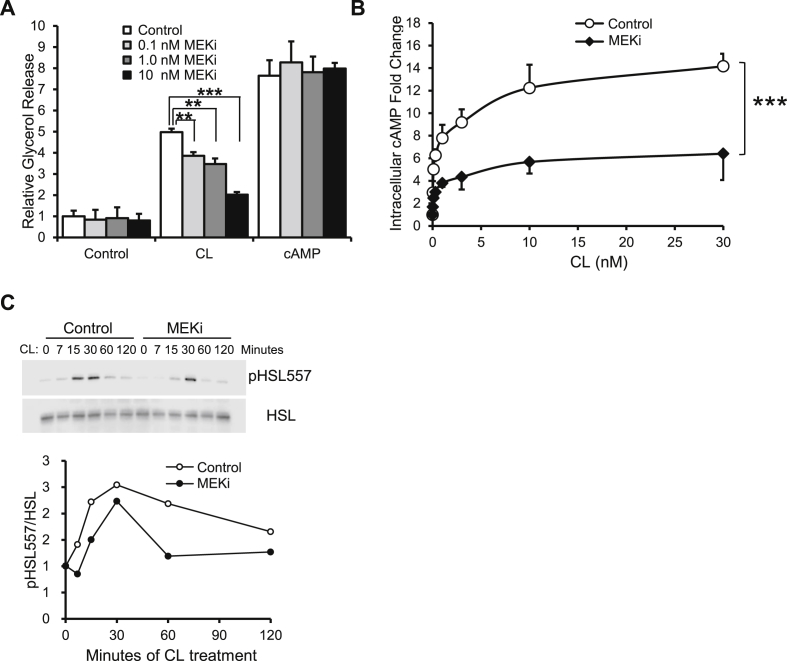
**Pharmacological inhibition of ERK phosphorylation suppresses β3AR signaling and lipolysis upstream of cAMP.** (A) Impact of different concentrations of MEKi (0 nM (Control), 0.1 nM, 1 nM and 10 nM MEKi) on basal (Control), CL induced (CL), and 8-Br-cAMP induced (cAMP) glycerol release from 3T3-L1 adipocytes. (B) Intracellular cAMP levels were measured 30 min after 3T3-L1 adipocytes were treated with indicated concentration of CL following pretreatment with 10 nM MEKi (MEKi, filled diamond) or pretreatment with DMSO vehicle control (Control, open circle). (C) Phosphorylation of HSL at Ser557 (pHSL557) and total HSL (HSL) levels in 3T3-L1 adipocytes at different time points after 1 μM CL treatment, with pretreatment of 10 nM MEKi or DMSO control. The intensity of each band from the western blot was analyzed and ratio of the intensities between pHSL557 and HSL were plotted and normalized by time point zero with DMSO treatment. Error bars represent SEM. Statistical significance was analyzed by one-way ANOVA (A) and two-way ANOVA (B). **, p < 0.01; ***, p < 0.005.

We next tested whether MEK inhibitors could impair β3AR agonist-induced lipolysis in a well-established cell line, 3T3-L1 adipocytes. Consistent with the observations from our *in vivo* and *ex vivo* experiments, CL induced lipolysis is diminished by MEK inhibitor treatment in a dose-dependent manner but cAMP-induced lipolysis is unaffected (Figure 5A). We confirmed MEKi treatment had no effect on adipocyte differentiation markers at any of the doses tested (Supplementary Fig. 5). Increases of intracellular cAMP levels and phosphorylation of hormone-sensitive lipase (HSL) are events downstream of β3AR activation. Thus, we monitored the intracellular levels of cAMP and phospho-HSL in 3T3-L1 adipocytes after the administration of CL and the MEK inhibitor. MEK inhibitor-treated cells had dramatically lower cAMP levels and weaker HSL phosphorylation compared to vehicle-treated control cells (Figure 5B,C).

### 3.10 Phosphorylation of β3AR at Ser247 is critical for β3AR signaling

**Figure 6:**
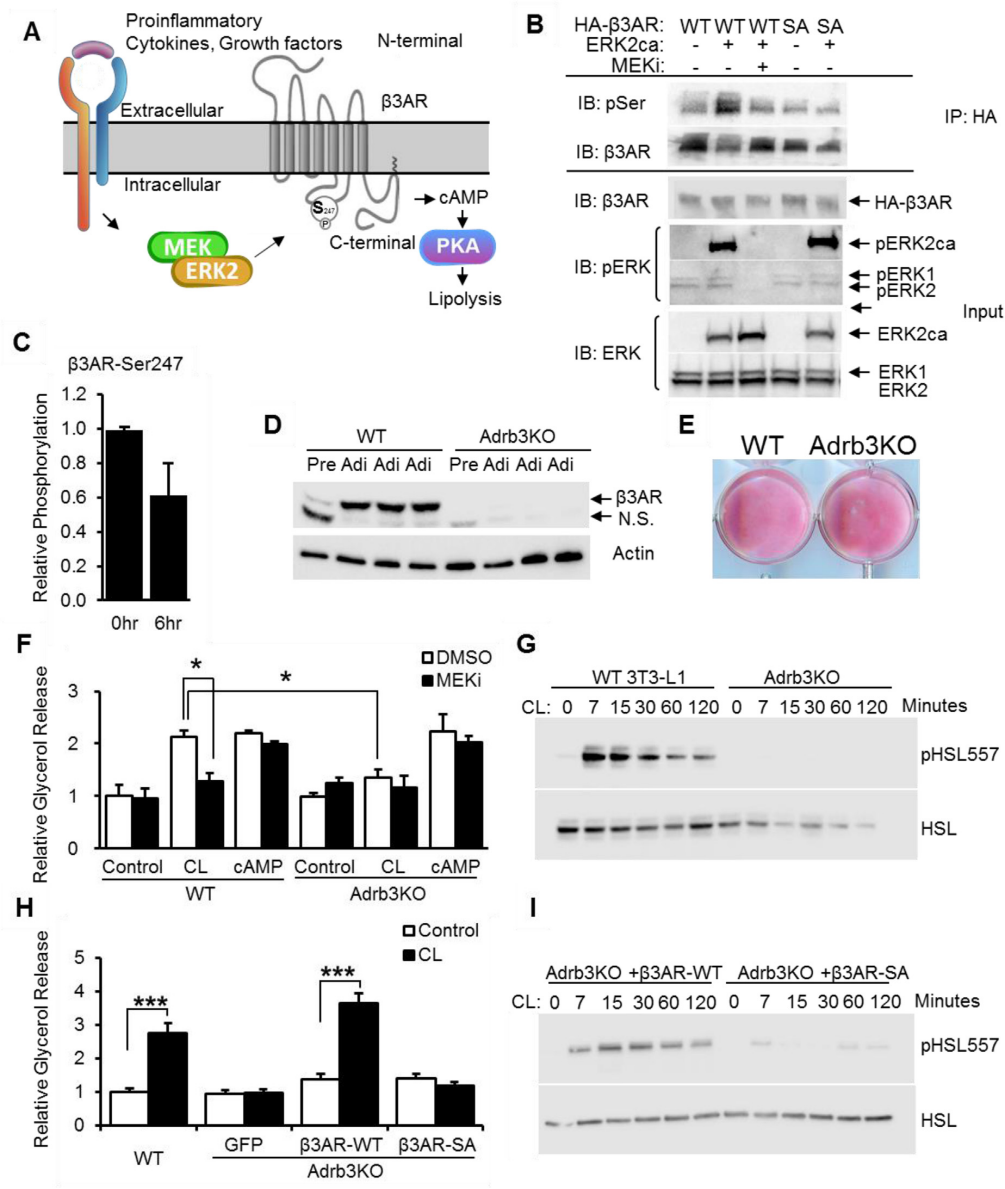
**Phosphorylation of β3AR at Ser247 is critical for β3AR signaling and lipolysis.** (A) Schematic depicting contribution of ERK signaling to phosphorylation of β3AR at Ser247 and impact on signaling and lipolysis. (B) Input and anti-HA co-immunoprecipitation from lysates of 293 T cells transfected with: HA-tagged wild type β3AR (WT), an HA-tagged β3AR mutant with Ser247 mutated to Ala (SA), and constitutively active ERK2 (ERK2ca), with or without treatment of MEK inhibitor (MEKi). Shown are immunoblots for β3AR and phosphorylated ERK substrate (pSer) following anti-HA immunoprecipitation, as well as expression of β3AR, phosphorylated ERK (pERK) and total ERK (ERK) in input protein lysates. (C) Relative phosphorylation of β3AR at Ser247 in mouse white adipose tissues 0 h and 6 h after the administration of MEK inhibitor, as detected by mass spectrometry. (D) β3AR and Actin protein expression in undifferentiated pre-adipocytes (Pre) and fully differentiated adipocytes (Adi) from wild type (WT) and β3AR deficient (Adrb3KO) 3T3-L1 cell lines. (E) Oil red O staining of differentiated wild type (WT) and β3AR deficient (Adrb3KO) 3T3- L1 adipocytes. (F) Relative glycerol release from 1 μM CL or 0.5 mM 8-Br-cAMP (cAMP) treated WT and Adrb3KO 3T3- L1 adipocytes, with pretreatment with 10 nM MEKi or DMSO control. (G) Phosphorylation of HSL at Ser557 and total HSL expression in CL treated WT and Adrb3KO adipocytes. (H) Relative glycerol release from WT 3T3-L1 (3T3-L1) adipocytes and Adrb3KO adipocytes overexpressing: GFP, wild type β3AR (β3AR-WT), β3AR mutant with Ser247 mutated to Ala (β3AR-SA), with (CL) or without (Control) 1 μM CL treatment. (I) Phosphorylation of HSL at Ser557 and total HSL expression in CL treated Adrb3KO adipocytes with overexpression of β3AR-WT or β3AR-SA. Error bars represent SEM. Statistical significance was analyzed by one-way ANOVA. *, p < 0.05; **, p < 0.01; ***, p < 0.005.

To explain the mechanism by which decreased ERK activation affects β3AR-induced lipolysis but not cAMP-induced lipolysis, we re-examined the phosphoproteomic results from *in vivo* MEK inhibition for upstream effectors (Figure 6A). Within the intermediate cluster (Figure 2B), we identified a phosphorylation site, serine 247, on the β3 adrenergic receptor. This site matches a non-canonical ERK site (PxxSP) predicted to have slower kinetics [47]. Two recent high throughput screens of 3T3-L1 adipocytes identified the existence of β3AR phosphorylation at this site, but neither study described the significance of this phosphorylation [48], [49]. Our results suggest that ERK phosphorylates β3AR on Ser247 and activates β3AR signaling, which, in turn, activates PKA and results in elevated lipolysis in adipocytes (Figure 6A). To test this model, we generated a hemagglutinin (HA) tagged β3AR mutant (β3AR-SA), whose serine at 247 was mutated to alanine. We then co-transfected this mutant or wild type β3AR (β3AR-WT), along with constitutively active ERK2 (ERK2ca), into 293 T cells. Wild type β3AR or mutated β3AR were purified by HA immuno-precipitation and immuno-blotted with an antibody specifically recognizing ERK substrates. β3AR-WT and β3AR-SA were expressed at similar levels in transfected 293 T cells (Figure 6B). However, only β3AR-WT and not β3AR-SA was phosphorylated by constitutively active ERK2, and this phosphorylation was blocked by MEK inhibitor treatment (Figure 6B), suggesting that ERK phosphorylates β3AR specifically at residue serine 247.

Phosphorylation of β3AR S247 was decreased by 40% in adipose tissue from obese mice 6 h following MEK inhibitor treatment (Figure 6C). Next, to test if phosphorylation of β3AR at Ser247 is necessary for activation of β3AR signaling, we generated a β3AR knockout 3T3-L1 cell line using CRISPR/CAS9 technology and attempted to use either β3AR-WT or β3AR-SA to rescue β3AR agonist-induced lipolysis. We designed a single guide RNA (sgRNA) targeting the first exon of *Adrb3* to disrupt the gene. Sequencing results of genomic DNA confirmed a cell line with a deletion of 112 base pairs, which contains part of exon 1 and intron 1 of the *Adrb3* gene (Supplementary Fig. 6A). Western blotting with a β3AR antibody confirmed the deficiency of β3AR in this β3AR knockout 3T3-L1 cell line before and after differentiation (Figure 6D). The β3AR null 3T3-L1 cells differentiated to mature adipocytes and accumulated lipid droplets similarly to control 3T3-L1 adipocytes, as indicated by the expression of adipocytes marker genes (Supplementary Fig. 6B) and Oil-O red staining (Figure 6E). Unsurprisingly, β3AR null adipocytes no longer respond to the β3AR-specific agonist CL to promote lipolysis (Figure 6F) or stimulate phosphorylation of HSL (Figure 6G). However, β3AR null 3T3-L1 cells respond normally to cAMP stimulation (Figure 6F). We then introduced a control protein (green fluorescent protein, GFP), β3AR-WT, or β3AR-SA to these β3AR null cells by lentivirus infection and generated cell lines with stable expression (Supplementary Fig. 6C). These cell lines expressed normal levels of adipocyte differentiation markers (Supplementary Fig. 6B). However, only the cells with overexpression of β3AR-WT, but not β3AR-SA, can rescue CL induced lipolysis (Figure 6H) and phosphorylation on HSL (Figure 6I), demonstrating that phosphorylation of β3AR at Ser247 is required for activation of β3AR signaling.

## 4 Discussion

### 4.1 ERK, obesity and lipolysis

Patients with visceral obesity have greater rates of basal lipolysis than lean controls [50], [51], [52], [53]. As lipolysis is an actively regulated enzymatic process, significant effort has focused on the factors driving increased FFA release. Obese adipose tissue becomes relatively inflexible and unresponsive to changing nutrient states. Specifically, in obesity, adipose tissue is resistant to the effects of insulin to inhibit lipolysis and promote additional energy storage as well as being resistant to the effect of catecholamines to stimulate lipolysis [51], [54], [55]. Hence, as adipose tissue mass increases, both rates of glucose uptake and lipolysis per additional kilogram are decreased. Recent efforts to define the mechanisms underlying catecholamine resistance have included differential regulation of adrenergic receptor protein levels, altered activity of the adipocyte phosphodiesterase PDE3B, and degradation of catecholamines by macrophages [55], [56], [57], [58]. Due to the competing effects of processes to decrease stimulated lipolysis and increased rates of basal lipolysis, obese mice and humans have roughly equivalent steady state levels of FFAs [59]. Despite the complicated regulation of FFA fluxes, inhibition of lipolysis improves insulin sensitivity [60]. In this study, we show that ERK directly controls rates of lipolysis *in vivo.* While we demonstrate that regulation of β3AR phosphorylation is one mechanism to explain the regulation of lipolysis, we also observed decreased phosphorylation levels of other ERK substrates following MEK inhibition (Supplemental Fig. 7). ERK substrate proteins which may impact lipolysis upstream of cAMP include the beta-adrenergic receptor kinase 1 (ADRBK1/GRK2), Adenyl cyclase 6 (ADCY6), and the Phosphodiesterases 4A and 4D (PDE4A, PDE4D). Other possible mechanisms may exist and will require additional study.

Increased lipolysis is one of the adverse sequelae of obesity but does not affect body weight *per se*. Inhibition of lipolysis by inhibiting MEK or HSL does not affect food intake, energy expenditure, or food absorption and consequently does not affect body weight [59]. Inhibition of ATGL similarly does not affect energy expenditure or food intake but decreases body weight by reducing intestinal energy absorption [12]. Treating obesity-induced lipolysis is likely to ameliorate systemic metabolic dysfunction but likely will not affect obesity.

### 4.2 Adipose tissue ERK kinase deficiency is not sufficient to perturb whole body insulin sensitivity

Following the discovery over 30 years ago that insulin signaling directly activates ERK [61], a consensus has emerged that ERK does not directly control insulin action on glucose uptake [24], [62], [63]. Further, unlike canonical insulin signaling, ERK is not subject to downregulation in obesity, i.e. “insulin resistance” does not appear to limit further activation of ERK in hyperinsulinemia, diabetes, and obesity (Figure 1C and [64], [65]). In contrast, there is abundant genetic evidence linking inflammation and activation of JNK MAP kinases with insulin resistance, though effective *in vivo* pharmacological agents have proven elusive [66]. In this context, we made the surprising discovery that whole body MEK inhibition *in vivo* strongly improves insulin sensitivity in obese mice. Treatment with Trametinib or PD0325901 improves whole body insulin sensitivity in *ob/ob* and diet-induced obese mice [5]. Both MEK inhibitors decrease lipolysis *in vivo* (Figure 2F). Understanding the key sites of ERK inhibitor action is complex, as it clearly acts on liver, kidney, pancreas, and other tissues but does not cross the blood-brain-barrier [58], [67], [68].

Genetic models can help clarify the tissue-specific effects of MEK inhibition. Here we demonstrate fat-specific deficiency of Drosophila ERK suppresses lipolysis, increases lipid storage, and decreases glycemic levels. Correspondingly, fat-specific overexpression of constitutively active ERK results in opposite phenotypes in *Drosophila*. We also show cell-autonomous effects of ERK inhibition to limit adipose tissue lipolysis *in vivo* in mice, in mouse adipose tissue and in human adipose tissue. Other studies have also examined the phenomenon of ERK regulation of adipocyte lipolysis. Greenberg et al. suggest that ERK can directly phosphorylate HSL at S600 to limit lipolysis [16]. We did not observe this site (now annotated as serine 591, Uniprot P54310-1) phosphorylated in our data set from obese mouse adipose tissue. If the primary mechanism of ERK action on lipolysis was through HSL S600 phosphorylation, MEK inhibition should have suppressed both β3AR agonist and cAMP-induced lipolysis; our observations showed the opposite result.

### 4.3 Hepatic contribution of ERK to metabolic homeostasis

Following adipose tissue ERK2 deletion, we observed localized improvement in insulin signaling in eWAT but not in iWAT or liver. Why does this difference not translate into whole body insulin sensitivity? It is likely that ERK action in multiple tissues cooperates with the effects observed here in adipose tissue to affect whole body insulin sensitivity. Although our observations on the effect of pharmacological MEK inhibition are fully consistent with the effects of genetic ERK2 deletion on adipose tissue lipolysis, the insulin sensitizing properties of whole body MEK inhibition were not recapitulated by adipose ERK2 deletion alone. Similarly, when made genetically obese, *Lep*^*ob/ob*^*::Erk1*^*−/−*^ mice exhibit drastically improved glucose homeostasis, despite similar body weight as leptin-deficient *Lep*^*ob/ob*^*::ERK1*^*+/+*^ controls; the mechanism for this finding has not been defined [69]. However, these effects must be independent of adipocytes as these cells express ERK2 and not ERK1 (Figure 4A). Previous reports strongly support a role for hepatic ERK in metabolism. Ectopic hepatic ERK activation in obese mice decreases triglyceride accumulation, promotes hyperglycemia and insulin resistance [70]. Intriguingly, decreased hepatic lipolysis in concert with increased lipogenesis may contribute to steatohepatitis [71]. *In vivo* pharmacologic MEK inhibition would reduce both adipose and hepatic lipolysis, while ERK2 adipose-specific genetic deletion is insufficient to improve insulin sensitivity. The effects of sub-chemotherapeutic doses of the MEK inhibitor Trametinib in healthy obese patients presents an area of future interest.

## 5 Conclusions

These studies provide important mechanistic insights to explain the long-established physiological observation that obese adipose tissue has higher rates of basal lipolysis. Increased lipolysis contributes to increased hepatic glucose production, hyperglycemia and type 2 diabetes. Obesity-induced inflammation and cytokine signaling increases ERK kinase activation and ERK substrate phosphorylation. In obesity, ERK phosphorylates β3AR in adipose tissue at S247 to increase rates of lipolysis (Figure 6A). The elevated flux of fatty acids adversely affects whole body insulin sensitivity [72]. Therapeutic efforts to decrease adipose tissue inflammation, lipolysis or MEK/ERK signaling would likely contribute to improved insulin resistance and decreased severity of type 2 diabetes.

## Author contributions

S.H. and A.S.B. devised the project, designed and performed experiments, and analyzed data. W.S. and N.P. performed fly work and assisted with manuscript preparation. P.H.Z. and B.L. assisted with sample preparation for *in vitro* and *in vivo* experiments; M.P.J, J.S., and S.P.G., designed, performed, and analyzed mass spectrometry phosphoproteomics experiments. A.I.M., Z.D., and D.C. performed indirect calorimetry experiments and assisted with analysis. J.A.H. and C.J.P. assisted with experimental interpretation; H.A., A.T., and L.L. provided human tissue and regulatory assistance. A.S.B. and S.H. wrote the manuscript with input from all authors.

## Appendix A Supplementary data

Online only supplemental materials include additional methods describing measurement of glucose homeostasis, gene expression, mass spectrometry data collection and analysis, western blotting, co-immunoprecipitation, isolation and differentiation of adipocytes, tissue fractionation, and assays of lipolysis and cAMP.

The complete data set of more than 9000 phosphorylated residues from DIO adipose tissue following *in vivo* MEK inhibition is included.

mmc1

mmc2

mmc3
