# Supplementary material for: Phosphorylation of Beta-3 adrenergic receptor at serine 247 by ERK MAP kinase drives lipolysis in obese adipocytes
Source: Mol Metab. 2018 Mar 29;12:25–38. doi: 10.1016/j.molmet.2018.03.012 (PMC6001906; doi:10.1016/j.molmet.2018.03.012)
Supplement: mmc1 [file mmc1.pdf]

Supplemental Methods

Glucose tolerance tests, insulin tolerance tests, and *in vivo* insulin signaling

DIO mice were generated by feeding mice a HFD which started at 5-7 weeks of age. Body mass was monitored weekly. Body composition of DIO mice was measured by EchoMRI at 18–20 weeks of HFD feeding. Glucose tolerance test (GTT) on HFD-fed mice was conducted after 13–15 weeks on HFD feeding. After GTT, mice were given one week to recover and were then subjected to insulin tolerance testing (ITT). As described previously, for GTT, HFD-fed mice were fasted overnight (16 h) and received 1 g/kg body weight glucose [1]. For ITT, mice were fasted for 4 h before receiving 1.5 U/kg body weight insulin. Blood glucose levels were monitored by glucometer strip readings of tail bleeds at 0, 15, 30, 60, 90 and 120 minute time points. Insulin signaling was performed by intraperitoneal injection of 4-hr fasted mice with PBS or human insulin at a dose of 10 units/kg body weight. At 10 minutes post injection, liver and WAT were harvested and snap frozen on liquid nitrogen.

Gene expression and qPCR

RNA from tissue or cell samples was isolated from TRI reagent using the Direct-Zol RNA miniprep kit (Zymo Research). cDNA was generated from 1 μg RNA by RT-PCR with Multiscribe Reverse Transcriptase and High-capacity cDNA Reverse Transcription Kit (Applied Biosystems). Quantitative PCR was performed using SYBR select Master Mix (Applied Biosystems) on a Light Cycler 480 II (Roche). Expression levels of target genes were assessed using the ΔΔCt method, and normalized by TBP or 36B4.

Western blotting

Snap frozen mouse tissues were disrupted using a TissueLyser (Qiagen) in RIPA lysis buffer containing 50 mM Tris-HCl (pH 7.5), 150 mM NaCl, 1% NP-40, 0.5% sodium deoxycholate, and 0.1% SDS. Lysis buffer was supplemented with HALT protease and phosphatase inhibitor cocktail (Thermo Fisher Scientific). Hypothalamus was collected using a 0.5 mm mouse coronal brain matrix. Cells were rinsed with ice-cold PBS and then collected in RIPA lysis buffer. Protein lysates were quantitated using Pierce BCA protein assay (Thermo Fisher Scientific). Western blotting was performed using Miniprotean TGx 4–20% SDS-PAGE gels, Tetra Cell rig, and Transblot Turbo transfer system (Biorad). Membranes were blocked with 5% milk in 1× TBS-T, before overnight incubation with primary antibodies. HRP-conjugated secondary antibodies against rabbit- and mouse-derived primary antibodies were used at 5000-fold dilution. Chemiluminescence was visualized, recorded digitally, and quantified using the Chemidoc XRS + Imaging System (Biorad Image Lab).

Co-immunoprecipitation of HA-tagged β3AR-WT and β3AR-SA

293T cells were transfected with pcDNA 3.1 vectors expressing HA-β3AR-WT or HA-β3AR-SA. Transfected cells were harvested and lysed in non-ionic coIP buffer (50 mM Tris HCl, 150 mM NaCl, 1% Triton X100, 1 mM EDTA) supplemented with 1× HALT cocktail (Thermo Fisher Scientific). Protein lysates were incubated with anti-HA affinity agarose rotating overnight at 4°C. The agarose beads were washed 3 times with coIP buffer before elution by 5 min incubation at 95°C with SDS sample buffer. Western blotting of co-immunoprecipitation eluates was performed as described above.

Isolation of SVF and primary adipocytes

Stromal vascular fraction (SVF) was generated from perigonadal or inguinal fat depots. Briefly, fat depots were dissected from euthanized 6–8 week old mice. Tissue was digested for 45 min shaking at 37°C in digestion medium consisting of PBS with 10 mM $CaCl_2$, 2.4U/mL Dispase (Roche), and 10 mg/mL collagenase D (Roche). Digestion reaction was terminated with growth medium consisting of high-glucose DMEM/F12 supplemented with penicillin/streptomycin and 10% FBS. The cell suspension was filtered through 70 μm and 40 μm cell strainers and centrifuged at 1,500 rpm for 5 minutes. The floating primary adipocytes were subjected to western blotting. The pelleted SVF cells were resuspended and plated in growth medium and expanded for 5–7 days before being re-plated for experiments.

Culture and differentiation of SVF and 3T3-L1
Isolated SVF cells and 3T3-L1 cells were cultured in 10% FBS supplemented F-12/DMEM and DMEM media, respectively, and allowed to reach 100% confluence. Two days later, media was removed and replaced with growth media supplemented with 5 µg/mL insulin, 0.5 mM Dexamethasone, 1 µM Rosiglitazone and 1 µM IBMX. Two days later, media was replaced with growth media supplemented with 5 µg/mL insulin and 1µM Rosiglitazone. The media was refreshed every two days with growth media until full differentiation.

Lipolysis assay
In 24-well plates, mouse or human adipose tissue explants or differentiated adipocytes were pretreated with drugs for 30 minutes and then washed with phenol red-free DMEM and treated with CL 316243 or 8-Br-cAMP at 37°C with shaking. Two hours after the treatment, 50 µL of the culture media were assayed for released glycerol with free glycerol reagent (F6428-40ML, Sigma-Aldrich).

cAMP assay
In 24-well plates, differentiated adipocytes were pretreated with indicated compounds for 30 minutes and then washed with phenol red-free DMEM and treated with CL-316,243. Thirty minutes after the treatment, the cells were collected in lysis buffer and assayed for cAMP level by AlphaScreen cAMP Assay Kit (6760635D, Perkin Elmer Life & Analytical Sciences), following the manufacturer's protocol and using a Perkin Elmer EnVision Plate Reader within the Harvard Institute of Chemistry and Cell Biology (ICCB)-Longwood.

Mass spectrometry data acquisition
C57Bl6/J male mice were obtained from Jackson Labs at 8 weeks of age and maintained on a 60% HFD until the group mean body weight exceeded 45 grams. Animals were placed into 5 groups of n=2 matching groups for similar mean body mass. For the 0, 0.5, 1, 6, and 12 hour oral treatments with 3 mg/kg Trametinib, all animals were fasted for 4 hours prior to sacrifice at noon. Epididymal white adipose tissue was harvested and snap frozen.

<u>Protein extraction and digestion</u>
Adipose tissues were mechanically lysed with a homogenizer with SDS lysis buffer (2.0 % SDS w/v, 250 mM NaCl, PhosStop (Roche, Madison, WI) phosphatase inhibitors, EDTA free protease inhibitor cocktail (Promega, Madison, WI) and 100 mM HEPES, pH 8.5). Lysates were reduced with 5 mM DTT and alkylated with iodoacetamide (14 mM) in the dark as previously described [2]. Protein was purified by methanol/chloroform precipitation. Protein pellets were resuspended in 8 M urea containing 100 mM HEPES (pH 8.5) and concentrations were measured by BCA assay prior to protease digestion. Protein lysates were diluted to 4 M urea and digested overnight with LysC (Wako, Japan) in a 1/100 enzyme/protein ratio. Protein extracts were diluted further to a 1.5 M urea concentration and trypsin (Promega, Madison, WI) was added to a final 1/200 enzyme/protein ratio for 6 hours at 37°C. Digests were acidified with 250 uL of 20% formic acid (FA) to a pH ~ 2 and subjected to C18 solid-phase extraction (SPE) (Sep-Pak, Waters, Milford, MA).

<u>Phosphopeptide enrichment</u>
Phosphopeptide enrichment was performed as previously described [3]. Briefly, peptides (~5 mg)

were resuspended in 1 mL of 2 M lactic acid/50 % acetonitrile (ACN) and pelleted at 15,000 g for 10 minutes. Supernatants were removed, placed in an Eppendorf tube containing 15 mg of titanium oxide beads (GL Sciences, Japan) and incubated for 1 hr. Beads were washed twice with 2 M lactic acid/50 % ACN and once with 0.1% TFA in 50 % ACN. Phosphopeptides were eluted twice with 150 uL of 50 mM HK_2PO_4, pH 10, acidified with 20 uL of 20% formic acid and subjected to C18 StageTip desalting (3M Empore, South Eagan, MN).

Tandem mass tagging (TMT) labeling

Isobaric labeling of the enriched phosphopeptides was performed using 10-plex tandem mass tag (TMT) reagents (Thermo Fisher Scientific). TMT reagents (5 mg) were dissolved in 252µl dry acetonitrile and 10 µl was added to ~100 µg of phosphopeptides dissolved in 100 µl of 200mM EPPS, pH 8.0. After 1hr, the reaction was quenched by adding 4 µl of 5% hydroxylamine. Labeled peptides were combined, acidified with FA (pH ~2) and diluted to a final ~1% ACN concentration prior to C_{18} SPE on Sep-Pak cartridges (50 mg).

Basic pH reversed-phase HPLC

TMT labeled phosphopeptides were subjected to orthogonal bpHrp fractionation. Labeled peptides were solubilized in buffer A (5% ACN 10 mM ammonium bicarbonate, pH 8.0) and separated by an Agilent 300 Extend C18 column (2.6 µm particles, 4.6 mm ID and 220 mm in length). Using an Agilent 1100 binary pump equipped with a degasser and a photodiode array (PDA) detector (Thermo Fisher Scientific, San Jose, CA), a 50 min linear gradient from 4% to 40% acetonitrile in 10 mM ammonium bicarbonate pH 8.0 (flow rate of 0.6 mL/min) separated the peptide mixtures into a total of 96 fractions. Fractions were consolidation into 12 samples, acidified with 10 ul of 20% formic acid and vacuum dried. Each sample was re-dissolved in 1% formic acid, desalted via StageTip, dried, and reconstituted for LC-MS/MS analysis.

Liquid chromatography separation and tandem mass spectrometry (LC-MS/MS) LC-MS/MS parameters

All spectra were acquired using an Oribtrap Fusion mass spectrometer (Thermo Fisher Scientific) in line with an Easy-nLC 1200 (Thermo Fisher Scientific) ultra-high pressure liquid chromatography (UHPLC) pump. TMT labeled peptides were separated onto a 75 µM inner diameter column containing 1 cm of Magic C4 resin (5 µm, 100 Å, Michrom Bioresources) followed by 45 cm of Sepax Technologies GP-C18 resin (1.8 µm, 120 Å) with a gradient consisting of 4–22% (ACN, 0.125% FA) over 180 min at ~300 nl/min. For all LC-MS/MS experiments, the mass spectrometer was operated in the data-dependent mode where the MS^1 spectra was set at a resolution of 120,000, with an AGC target of 150,000 and a max injection time of 75 ms. The ten most intense ions were selected for MS^2. MS^1 precursor ions were excluded using a dynamic window (90 seconds +/- 10 ppm) and the MS^2 precursors were isolated with a quadrupole mass filter set to a width of 0.5 DA. For MS^3 based TMT quantitation, MS^2 spectra were collected at an AGC of 4000, max injection time of 150 ms, and CID collision energy set at 35%. MS^3 spectra were acquired in the Orbitrap parameters where the HCD collision energy was increased to 55% at a resolution of 50,000. Synchronous-precursor-selection (SPS) was enabled to include up to 10 MS^2 fragment ions for the MS^3 spectrum.

Mass spectrometry analysis: Data processing and spectra assignment.

A compendium of in-house software was used to convert mass spectrometric data (Raw file) to a

mzXML format, as well as to correct monoisotopic m/z measurements and erroneous peptide charge state assignments. Assignment of MS/MS spectra was performed using the Sequest algorithm by searching the data against a protein sequence database including all entries the Mouse Uniprot database (download date June 3, 2015) containing known contaminants such as human keratins and its reverse decoy components [4]. Sequest searches were performed using a 10 ppm precursor ion tolerance and requiring each peptides N-/C- termini to have trypsin protease specificity, while allowing up to three missed cleavages. TMT tags on peptide N termini/lysine residues (+229.162932 Da) and carbamidomethylation of cysteine residues (+57.02146 Da) were set as static modifications while methionine oxidation (+15.99492 Da) and STY (+79.96633 Da) phosphorylation was set as variable modification. A MS^2 spectra assignment false discovery rate (FDR) of less than 1% was achieved by applying the target-decoy database search strategy [4]. Filtering was performed using an in-house linear discrimination analysis algorithm to create one combined filter parameter from the following peptide ion and MS^2 spectra metrics: Sequest parameters XCorr and ΔCn, peptide ion mass accuracy and charge state, peptide length and mis-cleavages. Linear discrimination scores were used to assign probabilities to each MS^2 spectrum for being assigned correctly and these probabilities were further used to filter the dataset to a 1% protein-level false discovery rate [2]. We used the Ascore algorithm to quantify the confidence with which each phosphorylation modification could be assigned to a particular residue in each peptide [5]. Phosphopeptides with Ascore values > 13 ($P \leq 0.05$) were considered confidently localized to a particular residue.

Determination of TMT reporter ion intensities and quantitative data analysis.
For quantification, a 0.003 *m/z* window centered on the theoretical *m/z* value of each ten reporter ions and the closest signal intensity from the theoretical *m/z* value was recorded. Reporter ion intensities were further de-normalized based on their ion accumulation time for each MS^3 spectrum and adjusted based on the overlap of isotopic envelopes of all reporter ions (as per manufacturer specifications). Total signal to noise values for all peptides were summed for each TMT channel, and all values were adjusted to account for variance in sample handling. For each peptide, a total minimum signal to noise value of 100 was required [6, 7]. Data were ranked by standard deviation, and the top 25% most variable peptides were retained for analysis. We performed fuzzy temporal clustering under the assumption that a peptide may belong to more than one group [8, 9]. Phosphorylation motif analysis was performed using Phosphosite Plus Sequence Logo Generator [10].

References for Supplemental Methods

[1] Palmer CJ, Bruckner RJ, Paulo JA, Kazak L, Long JZ, Mina AI, et al., 2017. Cdkal1, a type 2 diabetes susceptibility gene, regulates mitochondrial function in adipose tissue. Molecular metabolism.6(10):1212-25.

[2] Huttlin EL, Jedrychowski MP, Elias JE, Goswami T, Rad R, Beausoleil SA, et al., 2010. A tissue-specific atlas of mouse protein phosphorylation and expression. Cell.143(7):1174-89.

[3] Kettenbach AN, Gerber SA. 2011. Rapid and reproducible single-stage phosphopeptide enrichment of complex peptide mixtures: application to general and phosphotyrosine-specific phosphoproteomics experiments. Analytical chemistry.83(20):7635-44.

[4] Elias JE, Gygi SP. 2007. Target-decoy search strategy for increased confidence in large-scale protein identifications by mass spectrometry. Nature methods.4(3):207-14.

[5] Beausoleil SA, Villen J, Gerber SA, Rush J, Gygi SP. 2006. A probability-based approach for high-throughput protein phosphorylation analysis and site localization. Nature biotechnology.24(10):1285-92.

[6] McAlister GC, Huttlin EL, Haas W, Ting L, Jedrychowski MP, Rogers JC, et al., 2012. Increasing the multiplexing capacity of TMTs using reporter ion isotopologues with isobaric masses. Analytical chemistry.84(17):7469-78.

[7] Ting L, Rad R, Gygi SP, Haas W. 2011. MS3 eliminates ratio distortion in isobaric multiplexed quantitative proteomics. Nature methods.8(11):937-40.

[8] Aldridge BB, Saez-Rodriguez J, Muhlich JL, Sorger PK, Lauffenburger DA. 2009. Fuzzy logic analysis of kinase pathway crosstalk in TNF/EGF/insulin-induced signaling. PLoS computational biology.5(4):e1000340.

[9] Kumar L, Futschik ME. 2007. Mfuzz: a software package for soft clustering of microarray data. Bioinformation.2(1):5-7.

[10] Hornbeck PV, Kornhauser JM, Tkachev S, Zhang B, Skrzypek E, Murray B, et al., 2012. PhosphoSitePlus: a comprehensive resource for investigating the structure and function of experimentally determined post-translational modifications in man and mouse. Nucleic acids research.40(Database issue):D261-70.
