# Supplementary material for: Phosphorylation of Beta-3 adrenergic receptor at serine 247 by ERK MAP kinase drives lipolysis in obese adipocytes
Source: Mol Metab. 2018 Mar 29;12:25–38. doi: 10.1016/j.molmet.2018.03.012 (PMC6001906; doi:10.1016/j.molmet.2018.03.012)
Supplement: mmc3 [file mmc3.pdf]

Supplementary Figure 1

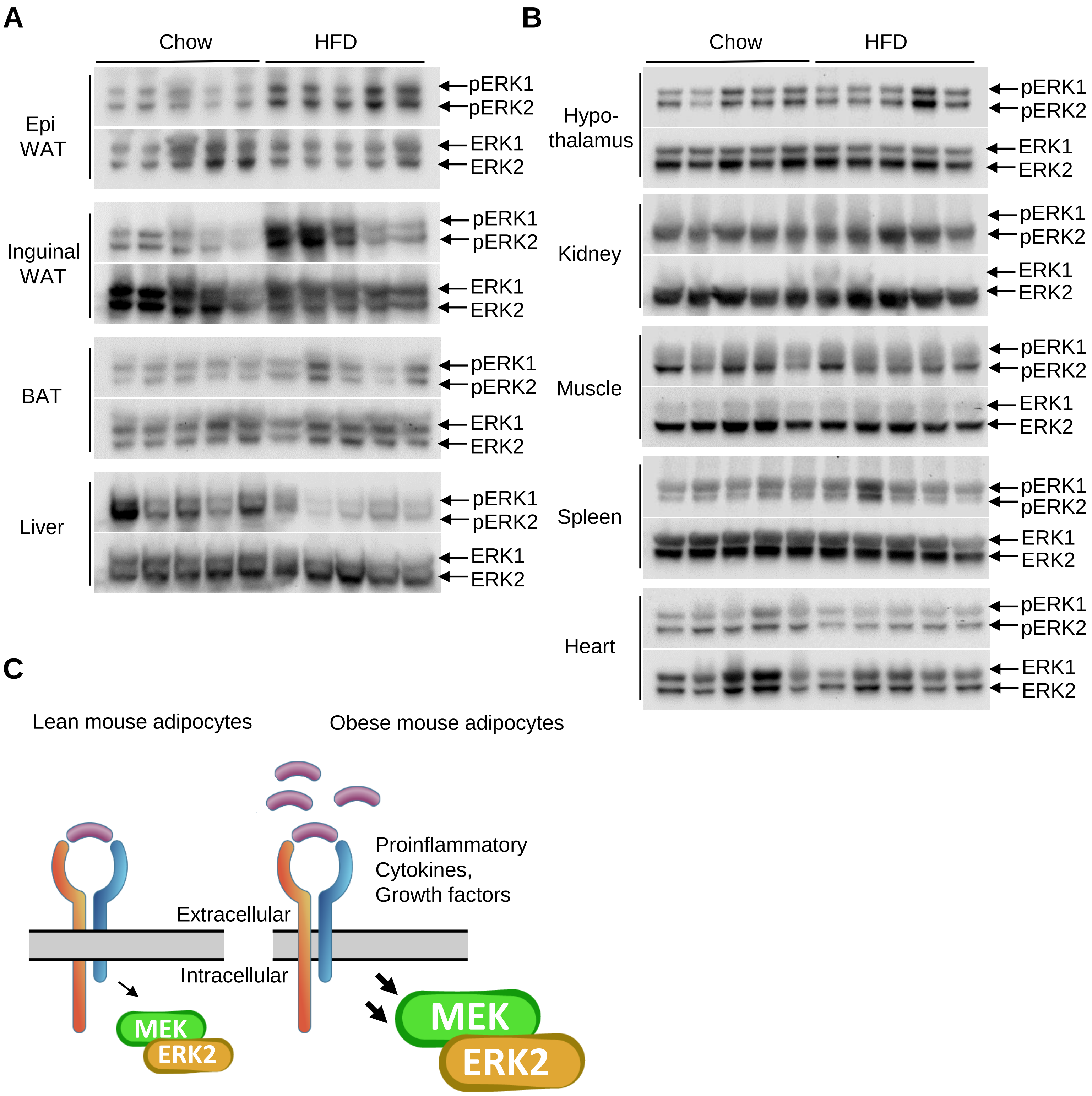

Supplementary Figure 1: Effect of obesity and insulin resistance on ERK phosphorylation. Western blotting of phosphorylated ERK1 and ERK2 relative to total ERK1 and ERK2 in (A) epididymal white adipose tissue (eWAT), inguinal white adipose tissue (iWAT), brown adipose tissues (BAT), and liver. (B) pERK/ERK levels in hypothalamus, kidney, muscle (quadriceps), spleen, and heart from age-matched 16 week old chow fed and HFD fed (8 weeks on HFD) mice. Mice were fasted for 4 hours prior to sacrifice. (C) Schematic depicting elevated ERK signaling attributed to obesity-induced pro-inflammatory cytokines in adipose tissue.

Supplementary Figure 2

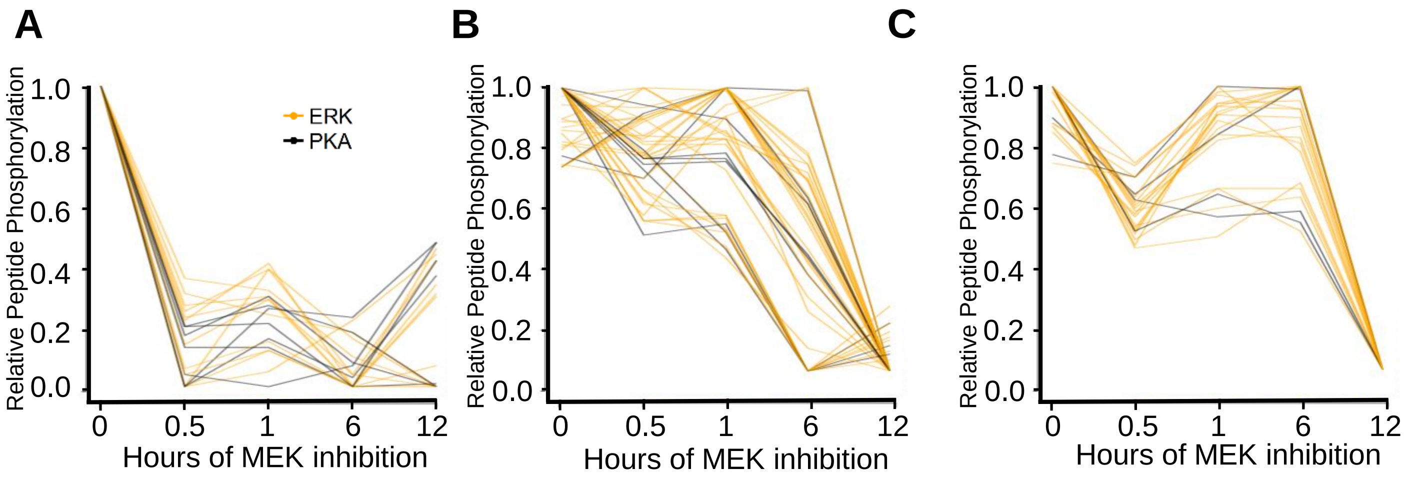

Supplementary Figure 2: MEK inhibition *in vivo* decreases phosphorylation of ERK and PKA substrates. (A-C) Temporal clustering of quantitative mass spectrometry on WAT phosphopeptides from diet induced obese mice following MEKi treatment for 0, 0.5, 1, 6, or 12 hours as in Figs 2A-C. Each line represents a unique peptide, and line color represents putative ERK phosphorylation motif (orange) or PKA (black) phosphorylation motif.

Supplementary Figure 3

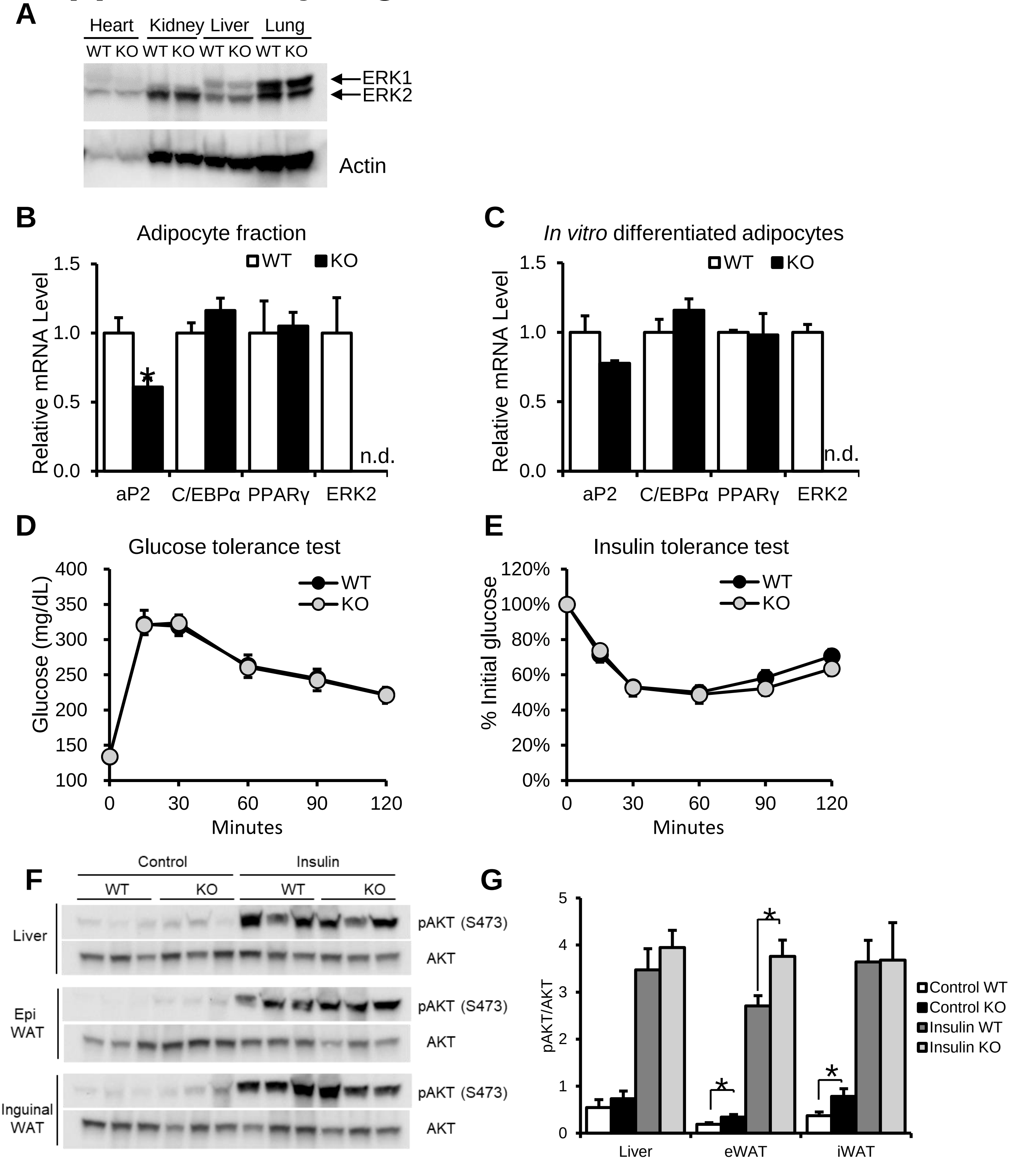

Supplementary Figure 3: Characterization of ERK2 AKO mice. (A) Protein levels of ERK1 and ERK2 levels in heart, kidney, liver and lung from wild type (WT) and ERK2AKO (KO) mice. (B-C) Expression of ERK2 and adipocyte marker genes (aP2, C/EBPα, PPARγ) from WT and ERK2AKO (KO) mice in (B) primary floated adipocytes (C) *in vitro* differentiated adipocytes derived from SVF (n=3 per genotype). (D) Blood glucose levels following IP-GTT on 13 to 15 weeks HFD-fed WT and ERK2AKO (KO) mice (n=9 per genotype). (E) Blood glucose levels following ITT (represented as % initial blood glucose levels) from 13 to 15 weeks HFD-fed WT and ERK2AKO (KO) mice (n=9 per genotype). (F) Insulin Signaling *in vivo*. Western blotting for phospho-AKT and total AKT after 4-hr fast (Control) or 4-hr fast ending with a bolus of insulin (Insulin). Tissues examined were liver, eWAT and iWAT.   n.d., not detected, Error bars represent SEM. *, $p < 0.05$, Student's t-test.

Supplementary Figure 4

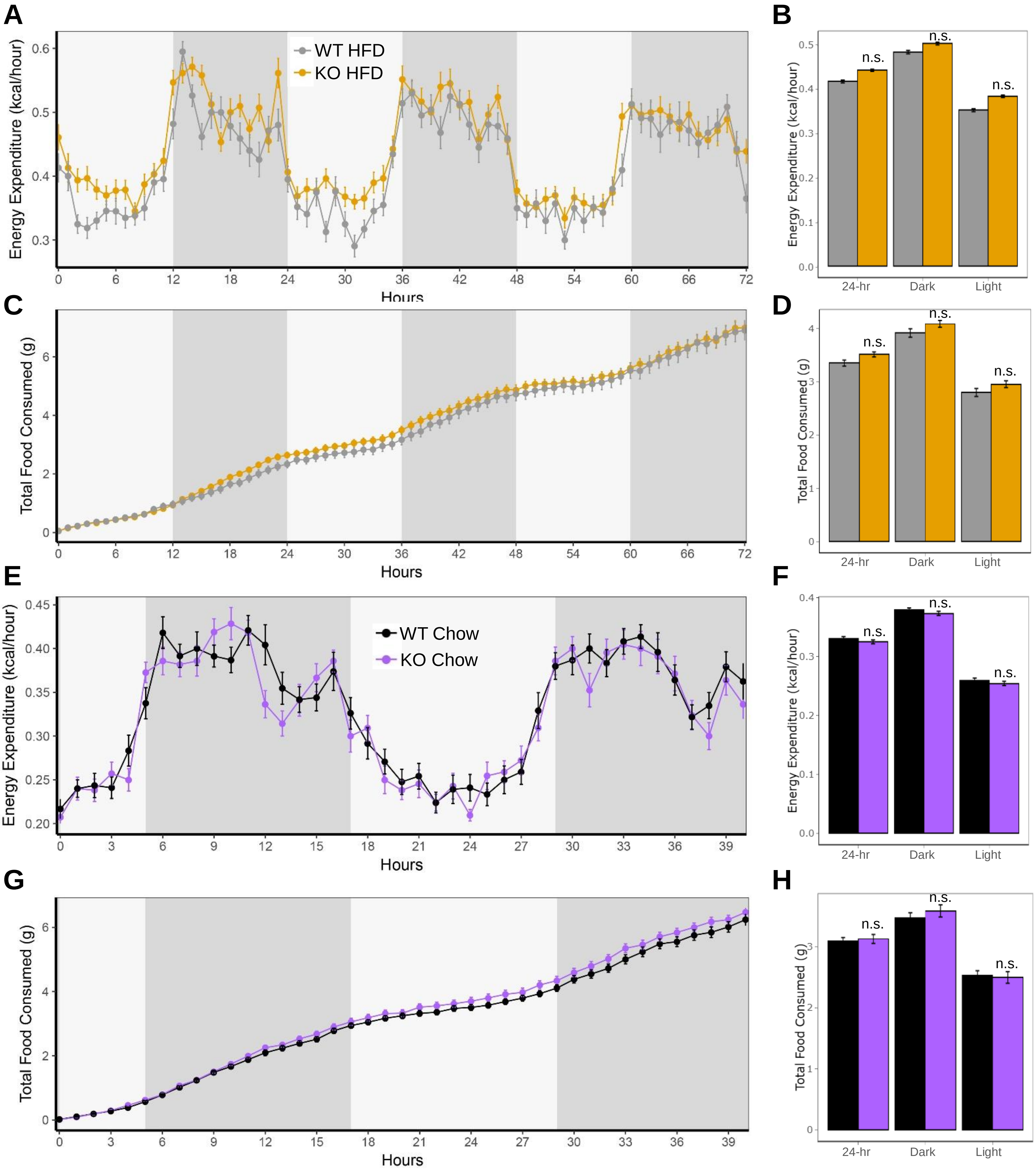

Supplementary Figure 4: Energy Balance in ERK2 AKO mice on standard or HFD. (A-D) Mice on HFD for 20 weeks were monitored for three days following acclimation. (A) Time plot of Energy Expenditure. (B) Overall 24-hr, dark and light photoperiod means are reported. (C) Time plot of food intake and (D) food intake overall and photoperiod means. n=9 male mice per genotype. (E-H) Mice on a standard chow diet for 16 weeks were monitored for two days following acclimation. (E-F) Energy Expenditure and (G-H) Food intake as above. n=8 male mice per genotype. All experiments were conducted at 30°C. Error bars represent SEM. Statistical analysis with *CalR* was used to perform ANCOVA using lean body mass as a covariate.

Supplementary Figure 5

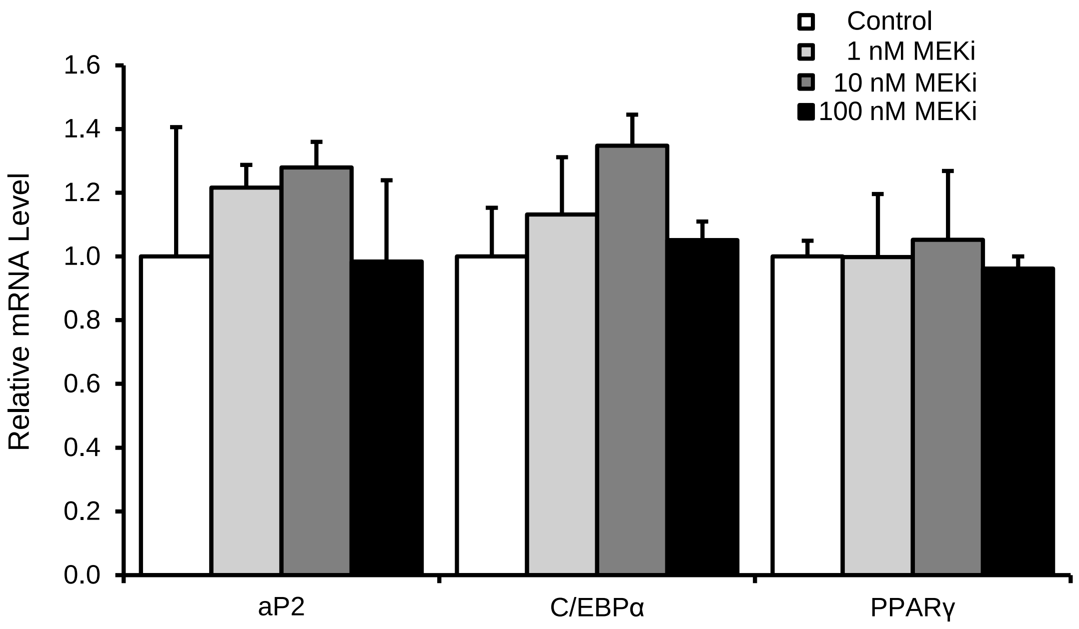

Supplementary Figure 5: MEKi treatment had no effect on adipocyte differentiation markers. Expression of adipocyte marker genes (aP2, C/EBPα, PPARγ) in fully differentiated 3T3-L1 adipocytes after overnight treatment with the indicated doses of MEKi.

Supplementary Figure 6

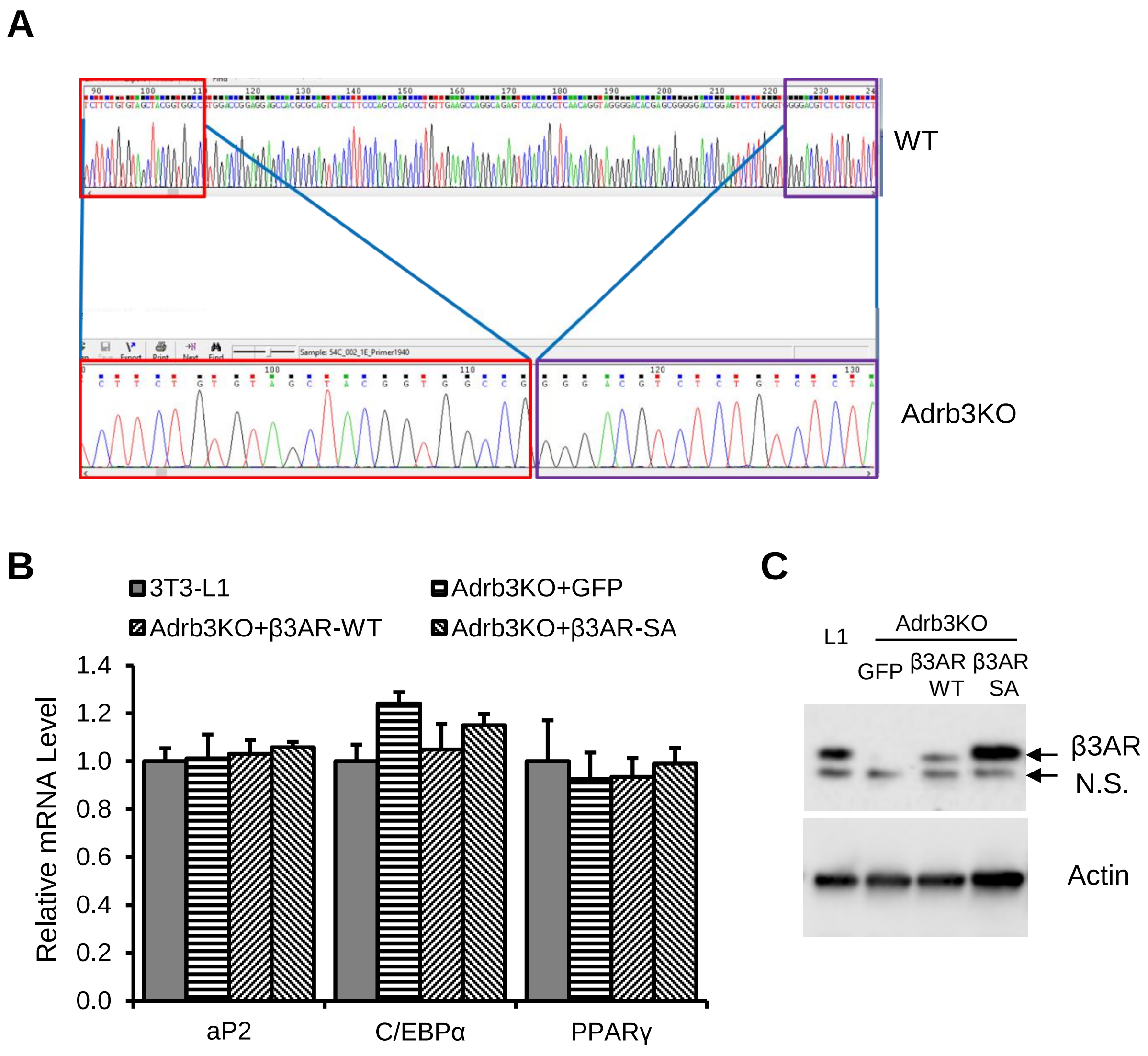

Supplementary Figure 6: Confirmation of β3AR deletion and overexpression in 3T3-L1 adipocytes. (A) Sequencing results of genomic DNA from wild type 3T3-L1 (WT) and β3AR knockout 3T3-L1 (Adrb3KO) confirmed a deletion of 112 base pairs (the fragment between the red box and the purple box) in β3AR knockout 3T3-L1 cell line, which contains part of exon 1 and intron 1 of the Adrb3 gene. (B) Expression of adipocyte marker genes (aP2, C/EBPα, PPARγ) in wild type 3T3-L1 adipocytes (3T3-L1) and β3AR null cells with overexpression of green fluorescent protein (Adrb3KO+GFP), wild type β3AR (Adrb3KO+β3AR-WT) or β3AR with Ser247-Ala mutation (Adrb3KO+β3AR-SA). (C) Endogenous and exogenous expression of β3AR in wild type 3T3-L1 adipocytes (L1) and Adrb3KO null cells with overexpression of green fluorescent protein (GFP), wild type β3AR (β3AR-WT), or β3AR with Ser247-Ala mutation (β3AR-SA).

Supplementary Figure 7

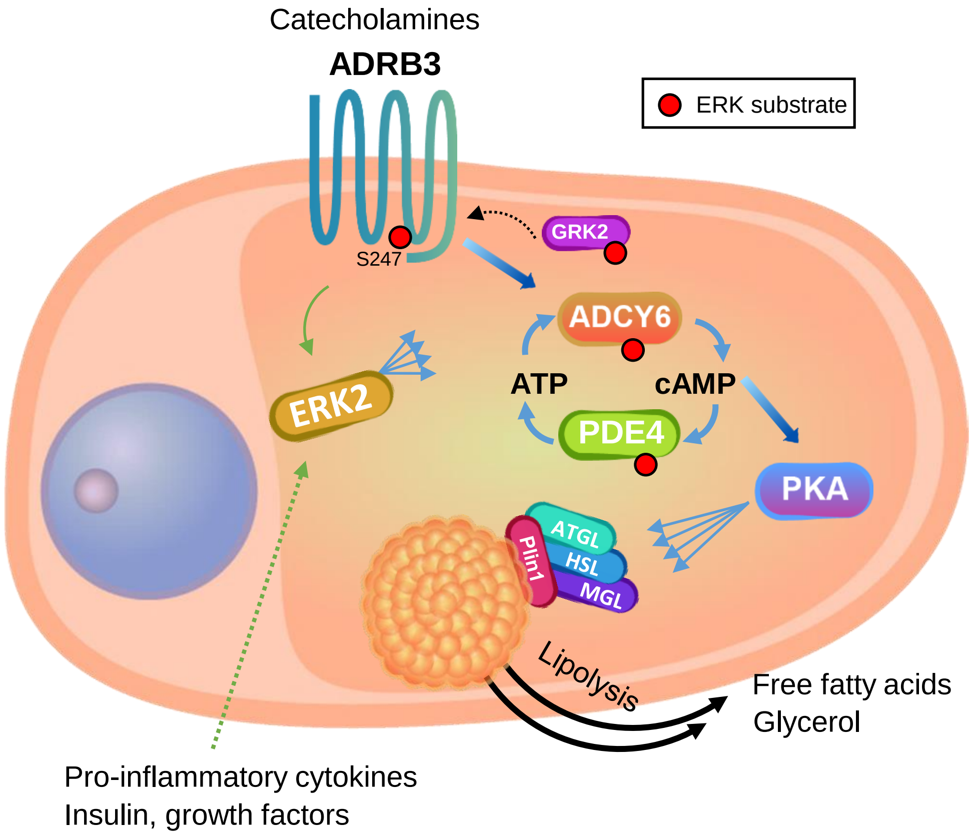

Supplementary Figure 7: Putative ERK substrates which may impact lipolysis. Schematic of proteins which may impact lipolysis due to ERK phosphorylation. ERK is activated by multiple signaling pathways in obese adipose tissue. The proteins listed have decreased phosphorylation levels following MEK inhibition at a possible ERK phosphorylation motif. Proteins are also known to affect cAMP levels or adrenergic signaling. Potential ERK target proteins include the ADCY6, ADRB3, GRK2/ADRBK1, and PDE4A/PDE4D. ADCY6 and PDE4 control levels of cAMP necessary for PKA activation. GRK2 may phosphorylate adrenergic receptors.
